# New insights in dehydration stress behavior of two maize hybrids using advanced distributed reactivity model (DRM). Responses to the impact of 24-epibrassinolide

**DOI:** 10.1371/journal.pone.0179650

**Published:** 2017-06-23

**Authors:** Hadi Waisi, Bojan Janković, Marija Janković, Bogdan Nikolić, Ivica Dimkić, Blažo Lalević, Vera Raičević

**Affiliations:** 1 Department of Scientific Research and Information Technology, Institute for the Development of Water Resources “Jaroslav Černi”, Belgrade, Serbia; 2 Department of General and Physical Chemistry, Faculty of Physical Chemistry, University of Belgrade, Belgrade, Serbia; 3 Radiation and Environmental Protection Department, Institute of Nuclear Sciences “Vinča”, University of Belgrade, Belgrade, Serbia; 4 Department for phytopharmacy and Environmental Protection, Institute for Plant Protection and Environment, Belgrade, Serbia; 5 Department of Microbiology, Faculty of Biology, University of Belgrade, Belgrade, Serbia; 6 Department for Environmental Microbiology, Faculty of Agriculture, University of Belgrade, Belgrade, Serbia; Universitat Zurich, SWITZERLAND

## Abstract

Proposed distributed reactivity model of dehydration for seedling parts of two various maize hybrids (ZP434, ZP704) was established. Dehydration stresses were induced thermally, which is also accompanied by response of hybrids to heat stress. It was found that an increased value of activation energy counterparts within radicle dehydration of ZP434, with a high concentration of 24-epibrassinolide (24-EBL) at elevated operating temperatures, probably causes activation of diffusion mechanisms in cutin network and may increases likelihood of formation of free volumes, large enough to accommodate diffusing molecule. Many small random effects were detected and can be correlated with micro-disturbing in a space filled with water caused by thermal gradients, increasing capillary phenomena, and which can induce thermo-capillary migration. The influence of seedling content of various sugars and minerals on dehydration was also examined. Estimated distributed reactivity models indicate a dependence of reactivity on structural arrangements, due to present interactions between water molecules and chemical species within the plant.

## Introduction

Maize is one of the most important cereals in the world both for human consumption and livestock feeding. Maize grain is used for all livestock production, while the whole maize plant is traditionally used for ruminants, mostly as silage [1]. Maize grain has a digestible energy content much higher than maize stover and it is assumed that the quality of forage maize is determined only by the ear to stover ratio, but this trait does not adequately predict the nutritional value of maize [2]. Due to its high water content, whole maize plant requires to be dehydrated before the inclusion in pelleted diets for some animals, and the maturity stage is important, since the dry matter content of maize plant increases from 23 to 37% during the maturing process of the grain [3].

In general, immature maize has a higher protein concentration than mature maize, but a lower energy value. As the grain progresses from early dough stage to commercial maturity, the percentage of grain in the plant increases, and consequently crude protein (CP) and crude fibre (CF) decrease while starch increases [4]. Maize dehydration is very important for its further distribution and storage as it is more hygroscopic than paddy rice and wheat and thus, absorbs moisture from the air more quickly.

A water deficit affects a broad range of plant functions, including growth, photosynthesis, metabolic pathways, and if severe enough, can cause tissue damage and death [5]. Plants respond to dehydration stress through physiological adjustments regulated by the expression of specific genes involved in the dehydration stress response [6,7]. Pre-exposure to diverse types of stresses, including dehydration stress, may alter subsequent responses, suggesting a form of “stress memory” [8,9]. Drought is likely the most important environmental factor that adversely affects plant growth and development [10,11,12]. The effects of drought on plants have been studied for a long time and changes induced by insufficient water supply have been examined from the whole plant/plant population level to the biochemical and molecular levels [13]. The primary and the most rapidly developing symptom of water stress in plants is a cessation of cell expansion caused by a decrease of turgor. Nevertheless, plants exhibit several adaptive strategies in response to various abiotic stresses such as salt, water, cold and dehydration stress, which ultimately affect plant growth and yield. To cope with these stresses, plants adapt various changes in physio-morphological and enzymatic processes [14].

Brassinosteroids (BRs) are capable of enhancing plant defense responce system against environmental stresses such as water, salt, heat, and cold stress [15,16]. Membrane stability and osmotic adjustment are two mechanisms through which brassinosteroids enhance abiotic stress tolerance [17,18,19,20,21]. Brassinosteroids (BRs) are a class of polyhydroxysteroids that have been recognized as a sixth class of the plant hormones. These hormones control and regulate various physiological processes in plants including cell differentiation, cell elongation, pollen tube development, swelling of cells, differentiation of vascular bundles, reassembling of nucleic acid to form proteins and acceleration of enzymatic processes as well as photosynthetic activities [22,23].

Aim of this work was to investigate the responses of particular seedling parts (radicle, plumule and the rest of the seed (RoS)) of two maize hybrids (ZP434 and ZP704) to dehydration at various and fixed operating temperatures through the setting of a newly proposed statistically derived interaction model. Actual processes were conducted in the presence and in the absence of the effects of different concentrations of 24-epibrassinolide (24-EBL). Special emphasis was given to identification of active centers (as bioactive compounds with functional chemical groups capable for release/binding of water molecules) in a given seedling parts during dehydration, which arise from derivation of the inherent distribution functions of activation energy counterparts (activation energy counterpart value is defined as the minimum energy necessary to take place an certain transformation, which may be physical or chemical origin).

## Materials and methods

### Materials

Different concentrations (5.20 × 10^−7^–5.20 × 10^−15^ M) of 24-epibrassinolide (active ingredient of the commercial preparation—“Epin-Extra”, obtained from GALENIKA-FITOFARMACIJA a.d. Zemun Company) were used in experiments. Two maize hybrids, ZP434 (drought tolerant) and ZP 704 (the older generation hybrid, which is standard hybrid, more susceptible to the stressful conditions) were tested. The seeds were produced in the “Maize Research Institute ZEMUN POLJE”, Republic of Serbia.

The seeds (4 × 50, whose weight was previously measured) were germinated in 2 L plastic boxes (each box contains 50 seeds), on filter paper sheets, topped at the beginning of the experiment with 60 mL of different concentrations of 24-EBL solution and under the phytothrone (Loške tovarne hladilnikov Škofja Loka, d.d., Slovenia) conditions at 24°C (over day) and 21°C (overnight), with a 12 hours of light (110–160 μmol photons m^-2^ s^-1^) / 12 hours of the dark regime [24]. After seven days, 4 × 25 uniformly grown seedlings from the boxes were measured using an analytical balance (Ohaus Pioneer, model PA413) and chosen for the thermal [25] and morphometric measurements.

### Sugar content determination

#### Preparation of sample test solutions

The method used for extraction and isolation of sugar compounds was a modification of the method originally developed for peach kernels analysis [26]. The fresh samples of seedling parts were placed into a previously heated oven (Carbolite Gero GmbH & Co. KG) at *T* = 130°C in an air atmosphere. Samples were tested at 130°C because that temperature does not affect most of the sugars. Approximately 0.25 g of maize sample (radicle, plumule, and the rest of the seed (RoS)) was suspended in 5 mL of ultra-pure water (Millipore Simplicity 185 S.A., 67120, Molsheim, France), the ultra-sonicated for 30 minutes, and centrifuged at 4000 rpm for 10 minutes upon *T* = 25°C. The supernatant was collected and the solid residue was re-extracted. The supernatants were combined, filtered through a 0.45 μm polytetrafluoroethylene (PTFE) membrane filter (Supelco, Bellefonte, PA, USA) and 1 mL of this solution was transferred into an auto-sampler vial. Individual sugars were determined and quantified using high performance anion-exchange chromatography with a pulsed amperometric detector (HPAEC-PAD). Sugar measurements were performed from three replicates.

#### Preparation of standard solutions

The calibration was performed with standard solutions of sugars, sorbitol (Sor) (Sigma–Aldrich, Steinheim, Germany), trehalose (Tre), fructose (Fru), sucrose (Suc), glucose (Glu), raffinose (Raf) (Tokyo Chemical Industry, TCI, Europe, Belgium). Each individual sugar standard was dissolved in ultra-pure water, and the stock solutions with a concentration of 1000 ng/mL were prepared. Dilution of the stock solutions with ultra-pure water yielded the working solutions at the concentration ranges that corresponded to the content of each sugar within the maize samples. The quality control mixture used for monitoring of instrument performance was prepared by diluting standards to the concentrations in the range 0.9–100 ng/mL (depending on the concentration in the samples).

#### HPAEC-PAD measurements

The chromatographic separations were performed using ICS 3000 DP liquid chromatography system (Dionex, Sunnyvale, CA, USA) equipped with a quaternary gradient pump (Dionex). The carbohydrates were separated on a Carbo Pac^®^PA100 pellicular anion-exchange column (4 × 250 mm, particle size—8.5 μm, pore size—microporous, < 10Å) (Dionex) at 30°C. The mobile phase consisted of the following linear gradient (flow rate, 0.7 mL/min.): 0–5 min., 15% A, 85% C; 5.0–5.1 min., 15% A, 2% B, 83% C; 5.1–12.0 min., 15% A, 2% B, 83% C; 12.0–12.1 min., 15% A, 4% B, 81% C; 12.1–20.0 min. 15% A, 4% B, 81% C; 20.0–20.1 min. 20% A, 20% B 60% C; 20.1–30.0 min. 20% A, 20% B 60% C, where A was 600 mM sodium hydroxide, B– 500 mM sodium acetate and C was ultra-pure water. Before the analyses, the system was pre-conditioned with 15% A, 85% C, for 15 minutes. Each sample (25 μL) with an ICS AS-DV 50 auto-sampler (Dionex) was injected. The electrochemical detector was consisted of gold as working and Ag/AgCl as reference electrode [26].

The LOD (Limit of detection) and LOQ (Limit of quantification) values were calculated using the standard deviation (SD) of the response and the slope (S) of the calibration curve of each analyte, according to the formulas: LOD = 3 × (SD/S) and LOQ = 10 × (SD/S). The values of SD and S were obtained from calibration curves created in the spreadsheet software of Microsoft Excel^®^ programs.

### Microwave digestion

A microwave-assisted acid digestion system (Berghof, Speed wave 4, Germany) was used to extract the elements from the samples (radicle, plumule and RoS). Approximately 0.50 g dry sample was digested. The digestion procedure was based upon recommendations by U.S. EPA guides for method 3051B [27] (with HNO_3_, HCl, and H_2_O_2_).

### ICP-OES determination

The solutions resulting from microwave digestion were analyzed using Spectro Genesis ICP-OES instrument with Smart Analyzer Vision software (SPECTRO Analytical Instruments GmbH, Boschstr. 10, 47533 Kleve, Germany). Curves were recorded on the basis of individual standards (Ultra Scientific, U.S.A. (concentrations of 1 g L^-1^)) and the multi standards (SPS-SW2, LGC, UK) for the targeted elements (Fe, P and K). The confirmation was carried out with three concentrations of matrix spike samples. Measurements were performed from three replicates.

### Isothermal measurements

The isothermal measurements were carried out in a high temperature oven Carbolite (Carbolite Gero GmbH & Co. KG) model LHT which operates in the temperature range from ambient up to 600°C, with a digital time counter and temperature-time graphic display. The oven comprising heating regime which is collinear with a maximum heating rate of 30°C per minute. The reaction atmosphere was air in a static pass. Thermal measurements of all hybrid samples were carried out at operating temperatures of *T* = 60, 105 and 130°C [25]. The seedlings parts from both maize hybrids (ZP434 and ZP704) were mechanically separated (by the cutting blades) and thus individually thermally treated (*c*_1_ = 5.20 × 10^−9^ M (60, 105 and 130°C), *c*_2_ = 5.20 × 10^−12^ M (60, 105 and 130°C), and *c*_3_ = 5.20 × 10^−15^ M (60, 105 and 130°C)). After reaching the desired operating temperature, about average mass in the range of 1.5566–8.7066 g (in respect of all concentrations of 24-EBL) for fresh radicle, plumule and RoS samples (the above mass represents the average cumulative sample mass from 25 uniformly compacted seedling parts) within ZP434 system, and about average mass in the range of 2.5469–8.6290 g (in respect of all concentrations of 24-EBL) also for the fresh radicle, plumule and RoS samples (where the above mass also represents the average cumulative sample mass from 25 uniformly compacted seedling parts) within ZP704 system, were placed in an oven under air conditions. Alumina crucibles were used in these experiments. The specimens were subjected to thermal treatment at constant operating temperatures between 60 and 130°C and durations between *t* = 8 min. and *t* = 30 min. After inserting the sample into the reaction oven block at the aimed temperature, the reaction time was followed by the computation. After that, the specimen was taken out of the oven and placed in the exicator. Each experiment at a given operating temperature was repeated three times. The weight results were noted as an average mass loss of the specimen.

The conversion fraction (or the extent of reaction, (α)) was calculated on the basis of the following equation: α = (*m*_o_—*m*_*t*_)/(*m*_o_—*m*_∞_), where *m*_o_ is the initial (at *t* = 0) mass of tested sample, *m*_*t*_ is actual mass of the sample at any other time than *t* = 0 (*t* ≠ 0), and *m*_∞_ is final (“equilibrium”) mass of the sample at *t* = ∞.

### Theoretical background

#### Distinctive distributed reactivity model

The first step in kinetic procedure is to obtain information about the reaction mechanism from the shape of the weight loss curve (thermo-analytical curve recorded under arbitrary heating programs). Where a single mechanism predominates, as in the case of gas phase or solution reactions, it is often possible to describe the reaction by a general rate law [28]:

$$\frac{d\alpha }{dt}=\underset{\begin{array}{l} Temperature-dependent\\ ratecons\text{tan}t\\ k\left(T\right) \end{array}}{\underset{︸}{A\text{ exp}\left(-\frac{E_{a}}{RT}\right)}}\cdot {\left(1-\alpha \right)}^{n}$$ (1)

where α is fraction converted (the extent of reaction), *t* is the time, *A* is the pre-exponential factor, *E*_*a*_ is the *effective* (apparent) activation energy, *R* is the gas constant, *T* is the absolute temperature, and *n* is the reaction order. Some relatively straight-forward solid-to- (solid + gas) reactions which may include the drying process within the biological system such as plant, we can therefore assume that the dehydration caused by thermal events occurs *via* first-order kinetics (which accurately describes the experimental results) including large number of independent, parallel, irreversible first-order chemical reactions with different activation energy counterparts (*ε*_*a*_) within unique *E*_*a*_ value (considering that the effective activation energy is the composite (the *complex*) magnitude, *E*_*a*_ = *ψ*_1_·*ε*_*a*1_ + *ψ*_2_·*ε*_*a*2_ + *ψ*_3_·*ε*_*a*3_ + … + ψ_*N*_·*ε*_*aN*_, where *ψ*_*i*_ represents *i*-th contribution of reaction with activation energy counterpart *ε*_*ai*_), reflecting variations in the bond strengths between water and host biomolecules or representing the energy intensity of the desorption centers, from which the water molecules evaporate and leave the tested biological system.

Following this postulate, the rate of water evolutions takes the following form:

$$\frac{d\alpha }{dt}=k\cdot \left(1-\alpha \right),$$ (2)

where *k* is the rate constant. When *k* follows the Arrhenius law, *k* = *A exp*(-*E*_*a*_/*RT*), Eq (2) can be transformed into an integral form after separation of variables and integration, as:

$$\frac{d\alpha }{\left(1-\alpha \right)}=A_{i}\int_{0}^{t}\text{exp}\left(-\frac{\sum_{i}\psi_{i}\cdot \varepsilon_{ai}}{RT}\right)dt,$$ (3)

where *A*_*i*_ is the pre-exponential factor related to corresponding *i*-th value of *ε*_*a*_. With the seed shape and higher relative moisture content (over 30%), maize dehydration is much more complex than with other seed crops, especially if the considered system is heated to a high operating temperatures, because many chemical species may participate in the interaction with the water molecules further hindering the evaporation of water. In this case, we can assume the existence of a probability density function *f*(*ε*_*a*_) of activation energy counterparts, where $\int_{\varepsilon_{a}}^{\varepsilon_{a}+\Delta \varepsilon_{a}}f\left(\varepsilon_{a}\right)d\varepsilon_{a}$ describes the probability that chemical species (or groups) within the sample have an activation energy counterpart between *ε*_*a*_ and *ε*_*a*_ + Δ*ε*_*a*_. The conversion fraction of evaporated material with activation energy counterparts between *ε*_*a*_ and *ε*_*a*_ + Δ*ε*_*a*_, at a given time *t*, is equal to:

$$d\alpha =f\left(\varepsilon_{a}\right)d\varepsilon_{a},$$ (4)

The passing differential (*d*) through the Eq (3), the equation for total conversion fraction (α) becomes:

$$\alpha (t)=1-\frac{f\left(\varepsilon_{a}\right)}{A_{i}\text{ exp}\left(-\frac{\sum_{i}\psi_{i}\cdot \varepsilon_{ai}}{RT}\right)\cdot t}d\varepsilon_{a},$$ (5)

where *T* [K] and *t* [min] represent the experimental operating temperature and active reaction time, respectively. The unknown parameters which need to be computed for each operating temperature (*T*) and all real values of *t* are as follows: *A*_*i*_, *ψ*_*i*_ and *ε*_*ai*_. Eq (5) represents a newly developed three-parameter conversion fraction relation for the investigation of dehydration processes for two maize hybrids tested in the actual paper. The values of *ε*_*ai*_ can be found for each individual and constant (fixed) fraction reacted value (α_*i*_ = *const*.), taking into account that *ε*_*a*_ is associated with *E*_*a*_. The procedure for requesting a functional dependence of *E*_*ai*_ on α_*i*_ (*E*_*ai*_ = *E*_*ai*_(α)) involves the use of *isoconversional* (“model-free”) methods [29]. These methods have ability to calculate *E*_*a*_ values without modelistic assumptions. In addition, these methods allow us to obtain *E*_*a*_−α reaction profiles, where we can draw conclusions about the general mechanistic scheme of the studied process.

From the integral form of Eq (2) (*g*(α) ≡ -ln(1-α) = *A* exp(-*E*_*a*_/*RT*) *t*, where *g*(α) is the integral form of the reaction mechanism function, assumed the first-order kinetics), and by the simple rearrangement of the obtained relationship, we can get the integral isoconversional equation in a form:

$$-\text{ln}t_{\alpha ,j}={\left[const.\right]}_{j}-\frac{E_{a,\alpha }}{RT_{j}},$$ (6)

where *t*_α,*j*_ is the time to reach a given fraction reacted at the different operating temperatures, *T*_*j*_. Thus, the value of the *effective* activation energy at a given α (*E*_*a*,α_) obtained by isoconversional method is determined by the slope of the plot of -ln*t*_α,*j*_ against 1/*T*_*j*_. To asses *E*_*a*_ − α dependence from isothermal data, the differential isoconversional equation can be used [30]. The differential isoconversional approach was based on Eq (2) in the logarithmic form such as:

$$\text{ln}{\left(\frac{d\alpha }{dt}\right)}_{\alpha ,j}=\text{ln}{\left[const.\right]}_{j}-\frac{E_{a,\alpha }}{RT_{j}}.$$ (7)

For constant α, the plot of ln(dα/d*t*)_α,*j*_ against 1/*T*_*j*_ obtained from the several isotherms should be a straight line whose slope allows to assess the *effective* activation energy value at a given α (*E*_*a*,α_).

Unfortunately, the density distribution function *f*(*ε*_*a*_) is unknown and its moments are inaccessible. However, it is possible to establish the experimentally evaluated density distribution function of activation energy counterparts (*f*_*exp*_[*E*_*a*_(*ε*_*a*_)]) from the functional relationship in a closed form:

$$f_{\text{exp}}\left[E_{a}\left(\varepsilon_{a}\right)\right]=\frac{d\alpha \left(E_{a}(\varepsilon_{a})\right)}{dE_{a}(\varepsilon_{a})},$$ (8)

where the current function represents the real experimentally derived density distribution function of effective energy values which appears during the complex process. The distribution of experimental points after the numerical derivative procedure dictates the shape of *f*_*exp*_[*E*_*a*_(*ε*_*a*_)] function, which may belong to the categories of discrete or continuous probability density functions, depending on the nature of the behavior of the observable random variable. The function expressed by Eq (8) is the versatile function which can be adapted in many circumstances (including abiotic plant stresses especially related to dehydration stress, where the plants largely depend on environmental factors) and, therefore, it was used therein. Usually, the *f*(*ε*_*a*_) function is assumed to have the form of the Gaussian distribution, but a disadvantage of the Gaussian distribution is that it is symmetric whereas the actual reactivity distributions in a complex processes tend to be asymmetric [31]. All numerical operations conducted in this paper have been used through application of computer program written in MATHEMATICA^®^ software (https://www.wolfram.com/mathematica/), which is capable to numerically solve the distributed reactivity model.

## Results

In the maize seedling samples of plumule, radicle and RoS, six sugars were identified and quantified, both for ZP434 and ZP704 hybrids. Sugars were examined for the whole concentration range of 24-EBL, as well as for control samples which are not treated with 24-EBL. Trehalose, glucose, raffinose, sucrose, fructose and sorbitol were chosen due to their role in theprotection and stabilization of biological molecules against various types of environmental stresses. From Table 1 we can notice that content of sugars varies with the change of 24-EBL concentrations at control temperature.

**Table 1:** Contents of various sugars determined in seedlings parts, with various concentrations of 24-EBL (5.20 × 10^−9^, 5.20 × 10^−12^ and 5.20 x 10^−15^ M) including control samples, for both studied hybrids.

**ZP434 hybrid (mg kg**^**-1**^ **of dry matter)**						
**Concentration**	**Raffinose**	**Sorbitol**	**Trehalose**	**Glucose**	**Fructose**	**Sucrose**
**Control** **Radicle**	7.9931	3.0579	62.3965	68.5418	254.3825	841.9138
**Control** **Plumule**	12.7217	3.3389	43.8622	222.6542	271.2674	649.1978
**Control** **RoS**	50.5664	5.5136	73.8872	1046.259	261.2864	130.0573
**5.20 × 10**^**−9**^ **Radicle**	14.2604	0.8227	32.2406	72.4003	110.0534	1210.0050
**5.20 × 10**^**−12**^ **Radicle**	7.0938	3.5868	22.2901	49.8711	114.6543	1338.2100
**5.20 × 10**^**−15**^ **Radicle**	5.0012	4.5679	29.6699	38.5214	178.4462	2169.5330
**5.20 × 10**^**−9**^ **Plumule**	17.0156	5.1357	115.7239	184.6312	252.1725	88.6012
**5.20 × 10**^**−12**^ **Plumule**	11.3800	1.4866	20.5768	41.0811	112.2853	2603.9130
**5.20 × 10**^**−15**^ **Plumule**	7.4408	1.1033	34.9129	212.4535	101.4513	2298.5210
**5.20 × 10**^**−9**^ **RoS**	34.1855	3.5327	84.7864	430.8231	117.0468	45.8835
**5.20 × 10**^**−12**^ **RoS**	32.8532	3.5152	226.9110	560.7511	116.8894	87.8414
**5.20 × 10**^**−15**^ **RoS**	21.9895	0.8227	333.7512	624.8609	162.6723	178.8451
**ZP704 hybrid (mg kg**^**-1**^ **of dry matter)**						
**Concentration**	**Raffinose**	**Sorbitol**	**Trehalose**	**Glucose**	**Fructose**	**Sucrose**
**Control** **Radicle**	6.8337	2.7508	84.8762	51.9034	61.4431	2527.6880
**Control** **Plumule**	28.7644	0.0059	53.2387	76.5831	110.2051	187.2433
**Control** **RoS**	27.8861	0.4146	92.7478	1042.7689	214.9874	46.5897
**5.20 × 10**^**−9**^ **Radicle**	45.3488	1.1700	73.8400	74.7900	69.5500	2206.3300
**5.20 × 10**^**−12**^ **Radicle**	34.9993	4.8500	217.1900	580.6900	585.6300	652.5600
**5.20 × 10**^**−15**^ **Radicle**	8.0947	0.6600	422.0900	101.2300	32.6200	2171.8300
**5.20 × 10**^**−9**^ **Plumule**	32.9978	3.1400	406.4000	1070.4500	602.9000	4642.3400
**5.20 × 10**^**−12**^ **Plumule**	76.9934	3.4400	264.4900	1054.3900	592.3500	4841.0200
**5.20 × 10**^**−15**^ **Plumule**	9.9465	5.5300	1002.3500	11.5700	26.5400	1148.2400
**5.20 × 10**^**−9**^ **RoS**	32.4110	1.6000	90.4700	845.1300	300.3500	137.6500
**5.20 × 10**^**−12**^ **RoS**	9.8344	0.2700	176.2900	773.3400	255.6000	49.6000
**5.20 × 10**^**−15**^ **RoS**	2.7681	0.2100	208.4300	175.2100	34.4500	4.7700

For the control samples attached to ZP434 maize hybrid, the raffinose content is the highest in RoS, whilst for ZP704 maize hybrid in the control samples, the raffinose is stored mostly in plumule and RoS (Table 1). In the case of ZP434 hybrid treated with different concentrations 24-EBL, the highest content of raffinose was identified in the radicle, plumule and RoS, at the highest exogenously added concentration of 24-EBL (5.20 × 10^−9^ M) (Table 1). On the other hand, for ZP704 hybrid, the highest content of raffinose was identified in the radicle and RoS at the highest exogenously added concentration of 24-EBL (Table 1), while in plumule, the raffinose is present in a greater content towards the “medium” exogenously added 24-EBL concentration (5.20 × 10^−12^ M) (Table 1).

For all considered cases (including both maize hybrids), the content of sorbitol is the lowest independent of the exogenously added concentration of 24-EBL, even in the case of the control samples.

When applying different concentrations of 24-EBL (Table 2), we can notice obvious differences in plumule and radicle emergences. There are differences in the length of plumule and radicle. In addition, there is evidence of inhibitory effect of exogenously added 24-EBL on seedling growth, which is noticeable at higher concentrations of the phytohormone [32].

**Table 2:** Principal dimensions (length, width and thickness) for the seed, plumule and radicle, including control samples and samples treated with various concentrations of 24-EBL (5.20 × 10^−9^, 5.20 × 10^−12^ and 5.20 × 10^−15^ M) for ZP434 and ZP704 hybrids, respectively.

**Control sample (mm)**	**ZP434**	**ZP704**
Seed length	9.1 ± 0.21 *	9.9 ± 0.08 *
Seed width	6.1 ± 0.08	6.3 ± 0.06
Seed thickness	3.8 ± 0.10	4.0 ± 0.10
Plumule length	41.2 ± 1.11	45.1 ± 1.16
Plumule width	3.2 ± 0.12	3.4 ± 0.08
Plumule thickness	1.1 ± 0.06	1.1 ± 0.06
Radicle length	186.0 ± 5.13	191.0 ± 5.77
Radicle width	1.1 ± 0.06	1.1 ± 0.06
Radicle thickness	1.1 ± 0.06	1.1 ± 0.06
**c = 5.20 × 10**^**−9**^ **24-EBL**		
**Sample (mm)**	**ZP434**	**ZP704**
Seed length	9.1 ± 0.08 *	9.9 ± 0.09 *
Seed width	6.1 ± 0.10	6.3 ± 0.06
Seed thickness	3.8 ± 0.13	4.0 ± 0.10
Plumule length	38.2 ± 0.40	42.2 ± 0.67
Plumule width	3.3 ± 0.12	3.2 ± 0.08
Plumule thickness	1.2 ± 0.06	1.3 ± 0.06
Radicle length	195.0 ± 2.75	186.0 ± 4.04
Radicle width	1.1 ± 0.06	1.1 ± 0.06
Radicle thickness	1.1 ± 0.06	1.1 ± 0.06
**c = 5.20 × 10**^**−12**^ **24-EBL**		
**Sample (mm)**	**ZP434**	**ZP704**
Seed length	9.1 ± 0.08 *	9.9 ± 0.13 *
Seed width	6.1 ± 0.13	6.3 ± 0.14
Seed thickness	3.8 ± 0.10	4.0 ± 0.06
Plumule length	38.2 ± 0.70	41.3 ± 0.49
Plumule width	3.3 ± 0.06	3.3 ± 0.10
Plumule thickness	1.2 ± 0.04	1.4 ± 0.06
Radicle length	195.0 ± 3.04	180.0 ± 3.79
Radicle width	1.1 ± 0.06	1.1 ± 0.06
Radicle thickness	1.1 ± 0.06	1.1 ± 0.06
**c = 5.20 × 10**^**−15**^ **24-EBL**		
**Sample (mm)**	**ZP434**	**ZP704**
Seed length	9.1 ± 0.10 *	9.9 ± 0.10 *
Seed width	6.1 ± 0.13	6.3 ± 0.08
Seed thickness	3.8 ± 0.10	4.0 ± 0.13
Plumule length	55.1 ± 1.62	42.2 ± 0.95
Plumule width	3.2 ± 0.13	3.2 ± 0.01
Plumule thickness	1.2 ± 0.03	1.1 ± 0.10
Radicle length	204.0 ± 4.62	178.0 ± 3.79
Radicle width	1.1 ± 0.06	1.1 ± 0.08
Radicle thickness	1.1 ± 0.06	1.1 ± 0.08

Asterisk represent “Standard-deviation for a sample” as a part of the population.

From presented results, we can see that examined concentrations had a stimulatory effect on hybrid ZP434 radicle length, while some concentrations had an inhibitory effect on hybrid ZP704 radicle length, comparing to control. It can be observed that lower concentration of exogenously added 24-EBL has a stimulatory effect on plumule length, for hybrid ZP434, while other concentrations have inhibitory effect on plumule length for hybrid ZP704, in comparison to control samples. The BRs interaction with other phytohormones causes modulation of gene expression, which results in plant growth such as cell elongation and elevation of resistance against different biotic and abiotic stresses [33].

From mineral composition analysis (Table 3), we can see that the levels (independent from influence of different 24-EBL concentration) of Fe, K and P are higher for ZP704 hybrid than those attached to ZP434 hybrid. For the control samples including all seedling parts, the level of Fe is more pronounced for ZP704 in comparison with ZP434 hybrid. However, the highest level of Fe is detected for the “most optimal” concentration of exogenously added 24-EBL (5.20 × 10^−12^ M) on the side of ZP704 hybrid (Table 3). In addition, at the lowest concentration of added 24-EBL (5.20 × 10^−15^ M) for plumule within ZP704, the level of Fe drops to zero, and the same phenomenon occurs for ZP434 within RoS at the highest concentration of added 24-EBL (5.20 × 10^−9^ M). The changes in Fe levels, especially for ZP434 hybrid, can be attributed to inhibition of the seed emergence, which may occur during the phosphorous limitation (Table 3). The highest level of potassium was detected in the case of ZP704 hybrid including radicle at highest concentration of added 24-EBL, but with a subsequent gradual decline in levels of K to the order of seedling parts (observing both maize hybrids).

**Table 3:** Mineral composition presents in the corresponding seedlings parts (radicle, plumule and RoS) with various concentrations exogenously added of 24-EBL (5.20 × 10^−9^, 5.20 × 10^−12^ and 5.20 × 10^−15^ M) including control samples, for both studied hybrids.

Concentration (M)	ZP434 hybrid (mg kg^-1^ of dry matter)			ZP704 hybrid (mg kg^-1^ of dry matter)		
	Fe	K	P	Fe	K	P
**Control** **Radicle**	1.1	1185.5	282.7	3.1	1588.2	1431.6
**Control** **Plumule**	13.3	979.7	770.0	33.2	1746.8	631.5
**Control** **RoS**	11.2	714.7	1090.7	30.7	598.2	946.7
**5.2 × 10**^**−9**^ **Radicle**	17.8	1761.3	225.9	11.2	3199.2	3065.0
**5.2 × 10**^**−9**^ **Plumule**	13.3	1412.8	350.1	10.7	1756.4	2362.1
**5.2 × 10**^**−9**^ **RoS**	0.0	482.2	412.0	25.2	1137.3	5424.7
**5.2 × 10**^**−12**^ **Radicle**	8.9	1435.6	131.6	60.5	1995.5	1772.2
**5.2 × 10**^**−12**^ **Plumule**	6.0	1334.4	212.8	23.1	1272.7	1982.9
**5.2 × 10**^**−12**^ **RoS**	3.8	450.7	381.0	121.9	1004.5	4564.7
**5.2 × 10**^**−15**^ **Radicle**	20.8	1428.8	104.3	27.5	1974.9	1968.7
**5.2 × 10**^**−15**^ **Plumule**	6.2	1214.8	213.1	0.0	1358.1	2015.6
**5.2 × 10**^**−15**^ **RoS**	11.0	326.7	316.4	57.7	448.0	3195.0

The highest level of phosphorus was observed in seedling parts of ZP704 hybrid at 5.20 × 10^−9^ M of exogenously added 24-EBL, and at 5.20 × 10^−12^ M of added 24-EBL for RoS (Table 3). It should be noted that such a large increase in the level of phosphorus in ZP704 monitors increase of potassium levels, while this behavior was not observed in control tests.

**Fig 1:**
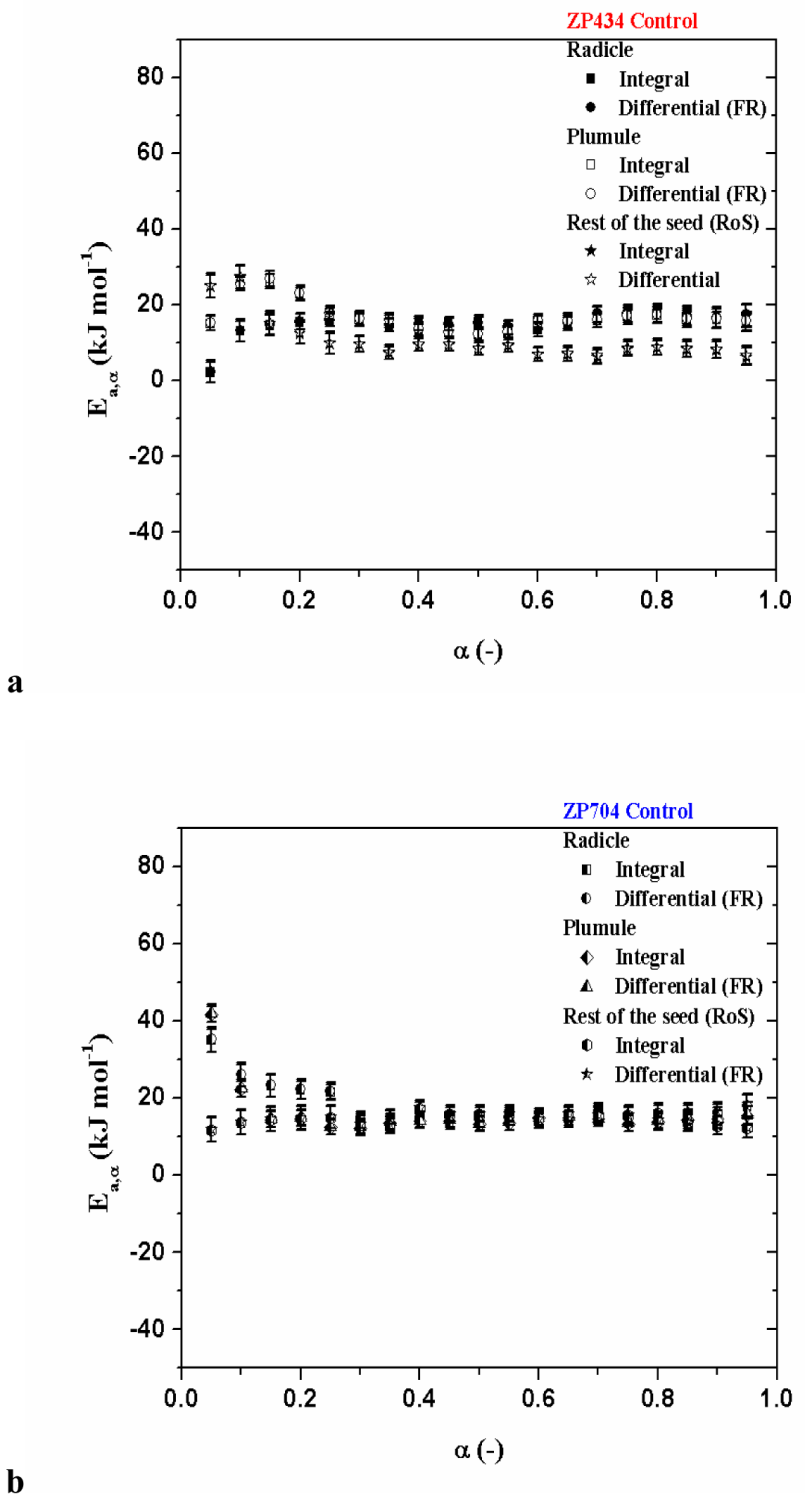
[(a)-(b)] Variation of apparent activation energies (*E*_*a*_) as a function of fraction reacted (α) for dehydration stress, determined by integral and differential (Friedman’s) isoconversional methods, for control tests within all seedling parts attached to ZP434 and ZP704 hybrids, respectively.

The shape of *E*_*a*_ dependence on α including all seedling parts for ZP434 and ZP704 hybrids is not the same, and show a different behavior, where field of constancy of *E*_*a*_ values do not occupy the same regions (Fig 1a and 1b). It is obvious that the mechanistic pictures of dehydration stress of these two hybrids are not identical. The increase in *E*_*a*_ value, in the initial stage of the process, for both maize hybrids including all seedling parts (except for RoS in ZP704) (Fig 1a and 1b), suggests that the dehydration does not follow a specific mechanism pathway.

**Fig 2:**
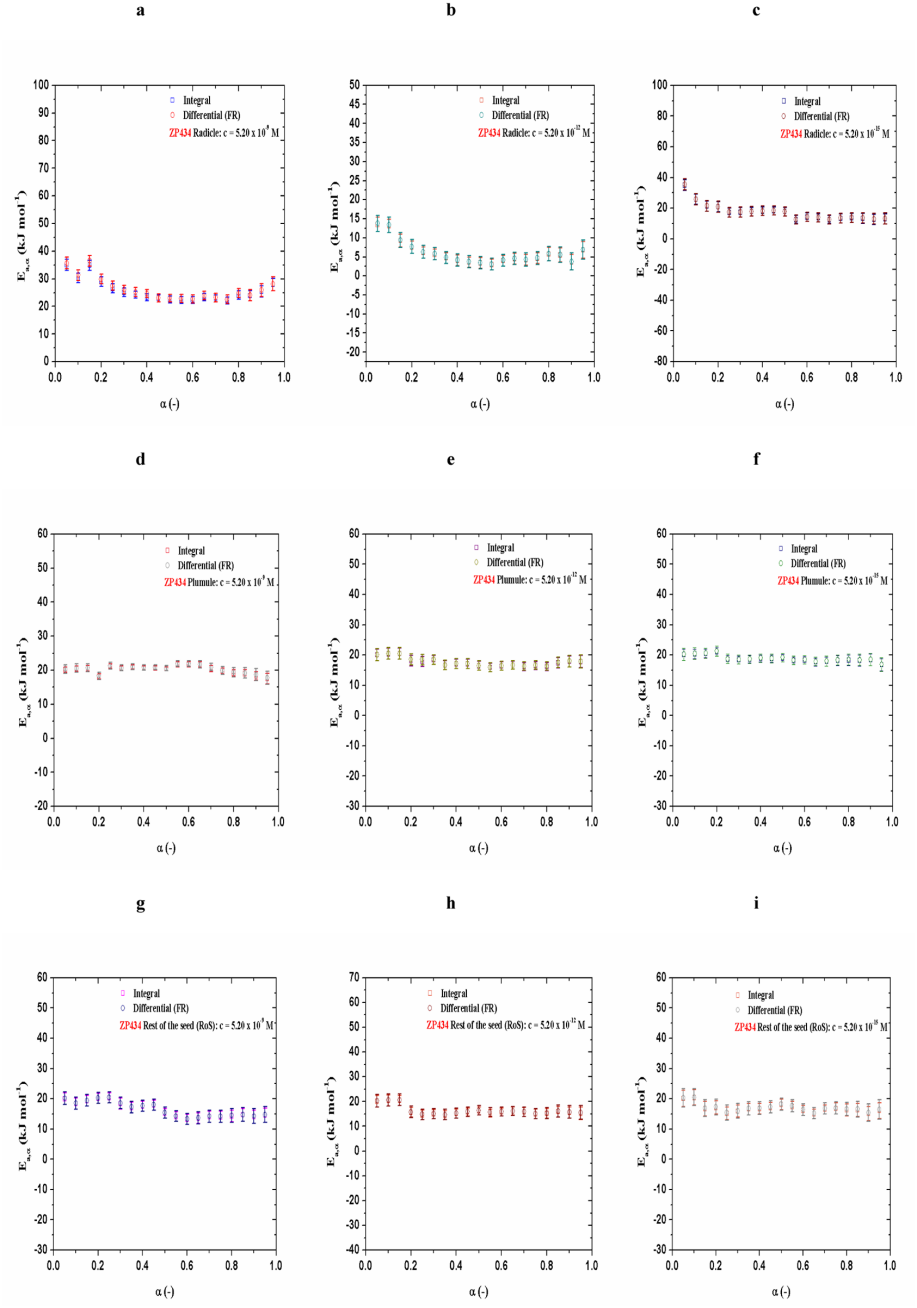
[(a)-(i)] Variation of apparent activation energies (*E*_*a*_) as a function of α, determined by integral and differential isoconversional methods, for ZP434 seedling parts related with different 24-EBL concentrations (5.20 × 10^−9^ M, 5.20 × 10^−12^ M, and 5.20 × 10^−15^ M).

**Fig 3:**
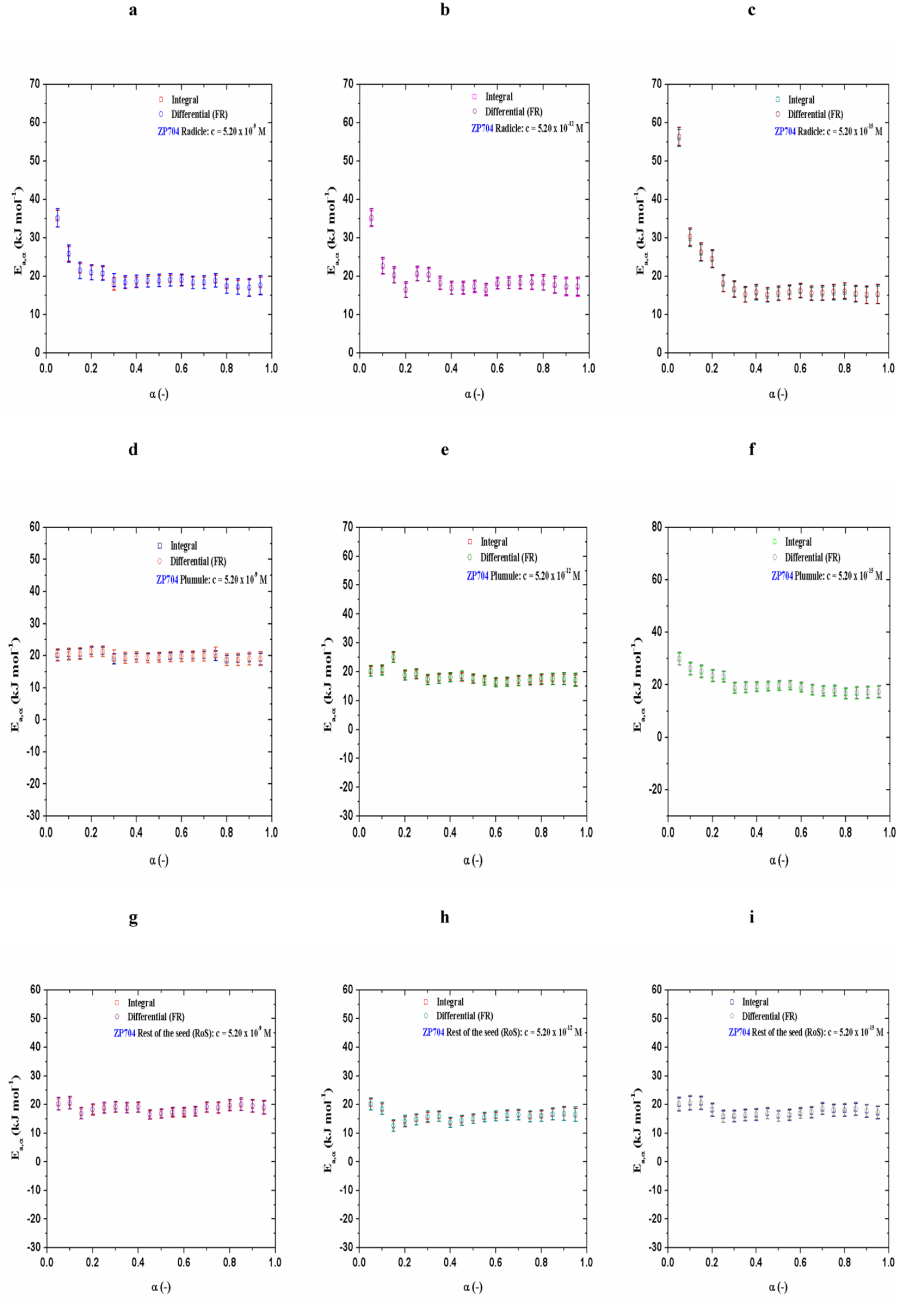
[(a)-(i)] Variation of apparent activation energies (*E*_*a*_) as a function of α, determined by integral and differential isoconversional methods, for ZP704 seedling parts related with different 24-EBL concentrations (5.20 × 10^−9^ M, 5.20 × 10^−12^ M, and 5.20 × 10^−15^ M).

The initial stages of dehydration reactions are often complex and involve several processes with different dependencies on time. However, in both cases (Fig 1a and 1b), in the regions in which *E*_*a*_ is nearly constant [ZP434 (control), Fig 1a: a) Radicle: <*E*_*a*_>_Int_ = 15.7 ± 1.9 kJ mol^-1^, <*E*_*a*_>_FR_ = 15.9 ± 2.1 kJ mol^-1^ (for 0.20 ≤ α ≤ 0.90), b) Plumule: <*E*_*a*_>_Int_ = 15.1 ± 1.8 kJ mol^-1^, <*E*_*a*_>_FR_ = 15.3 ± 2.0 kJ mol^-1^ (for 0.30 ≤ α ≤ 0.90), c) RoS: <*E*_*a*_>_Int_ = 8.4 ± 1.9 kJ mol^-1^, <*E*_*a*_>_FR_ = 8.6 ± 2.1 kJ mol^-1^ (for 0.25 ≤ α ≤ 0.90); ZP704 (control), Fig 1b: a) Radicle: <*E*_*a*_>_Int_ = 14.9 ± 2.1 kJ mol^-1^, <*E*_*a*_>_FR_ = 15.1 ± 2.3 kJ mol^-1^ (for 0.30 ≤ α ≤ 0.90), b) Plumule: <*E*_*a*_>_Int_ = 13.9 ± 2.0 kJ mol^-1^, <*E*_*a*_>_FR_ = 14.2 ± 2.2 kJ mol^-1^ (for 0.25 ≤ α ≤ 0.90), c) RoS: <*E*_*a*_>_Int_ = 14.9 ± 2.2 kJ mol^-1^, <*E*_*a*_>_FR_ = 15.2 ± 2.3 kJ mol^-1^ (for 0.15 ≤ α ≤ 0.85)], dehydration process can be assumed to follow a single step dehydration. For samples treated with 24-EBL, their *E*_*a*_ = *E*_*a*_(α) dependencies (Fig 2a–2i) and (Fig 3a–3i) show variations which are different from those identified in control samples (Fig 1a and 1b).

For ZP434 hybrid, decline of concentration presents of 24-EBL leads to some changes in *E*_*a*_ = *E*_*a*_(α) dependencies for radicle dehydration, wherein the “medium” exogenously added concentration (5.20 × 10^−12^ M) sustainable value of *E*_*a*_ at lowest level, unlike other two concentrations (Fig 2a–2c). Variation of *E*_*a*_ with α has lowest magnitude at lowest concentration of added 24-EBL for radicle dehydration (Fig 2c).

For same maize hybrid, for plumule dehydration, *E*_*a*_ = *E*_*a*_(α) profiles at all monitored added concentrations of 24-EBL are nearly identical, with a smaller rate of variability (show very similar values of the apparent activation energy (*E*_*a*_)) than those identified for radicle (Fig 2d–2f).

In the case of RoS, the similar behavior in relation to a change of *E*_*a*_ with α as in the case of the plumule can be identified (with similar values of the apparent activation energy), but with a higher degree of variation of *E*_*a*_ with α, especially at the beginning of the dehydration process (Fig 2g–2i).

However, ZP704 maize hybrid is shown in a somewhat different light than the previous one in terms of the variation of apparent activation energy values with conversions for dehydration process. Namely, radicle showed almost identical shape of *E*_*a*_ = *E*_*a*_(α) dependency at all added concentrations of 24-EBL (Fig 3a–3c) as dependence of *E*_*a*_ on α in the case of radicle for control test (Fig 1b). In this sense, the presence of concentration levels of exogenously added phytohormones does not affect the isoconversional dehydration profile for radicle attached to ZP704.

In the case of plumule, the presence of higher and medium concentrations of exogenously added 24-EBL (Fig 3d and 3e) stabilize the values of *E*_*a*_, compared to those identified in the control test (Fig 1b). The presence of a low concentration of added 24-EBL (5.20 × 10^−15^ M; Fig 3f) leads to a repeated increase in *E*_*a*_ values at the beginning of the dehydration process, as in the case of the control test (Fig 1b). In addition, the presence of various concentrations of added 24-EBL in the RoS (Fig 3g–3i) does not lead to a drastic variation in *E*_*a*_, so that the dependencies of *E*_*a*_ on α are very similar to those observed the case of RoS for the control test attached to ZP704 hybrid (Fig 1b). However, slightly increased amplitude of variation of the apparent activation energy was observed at beginning of process up to 20% of reacted fraction at highest and “medium” concentrations of applied 24-EBL (5.20 × 10^−9^ M and 5.20 × 10^−12^ M) (Fig 3g and 3h).

We can observe that presence of added 24-EBL has the greatest impact on the change of the isoconversional reaction profiles for the radicle and plumule dehydration processes attached to ZP434 hybrid, as well as on isoconversional profile for plumule dehydration process attached to ZP704 hybrid at 5.20 × 10^−9^ and 5.20 × 10^−12^ M of 24-EBL (Figs 2 and 3).

**Fig 4:**
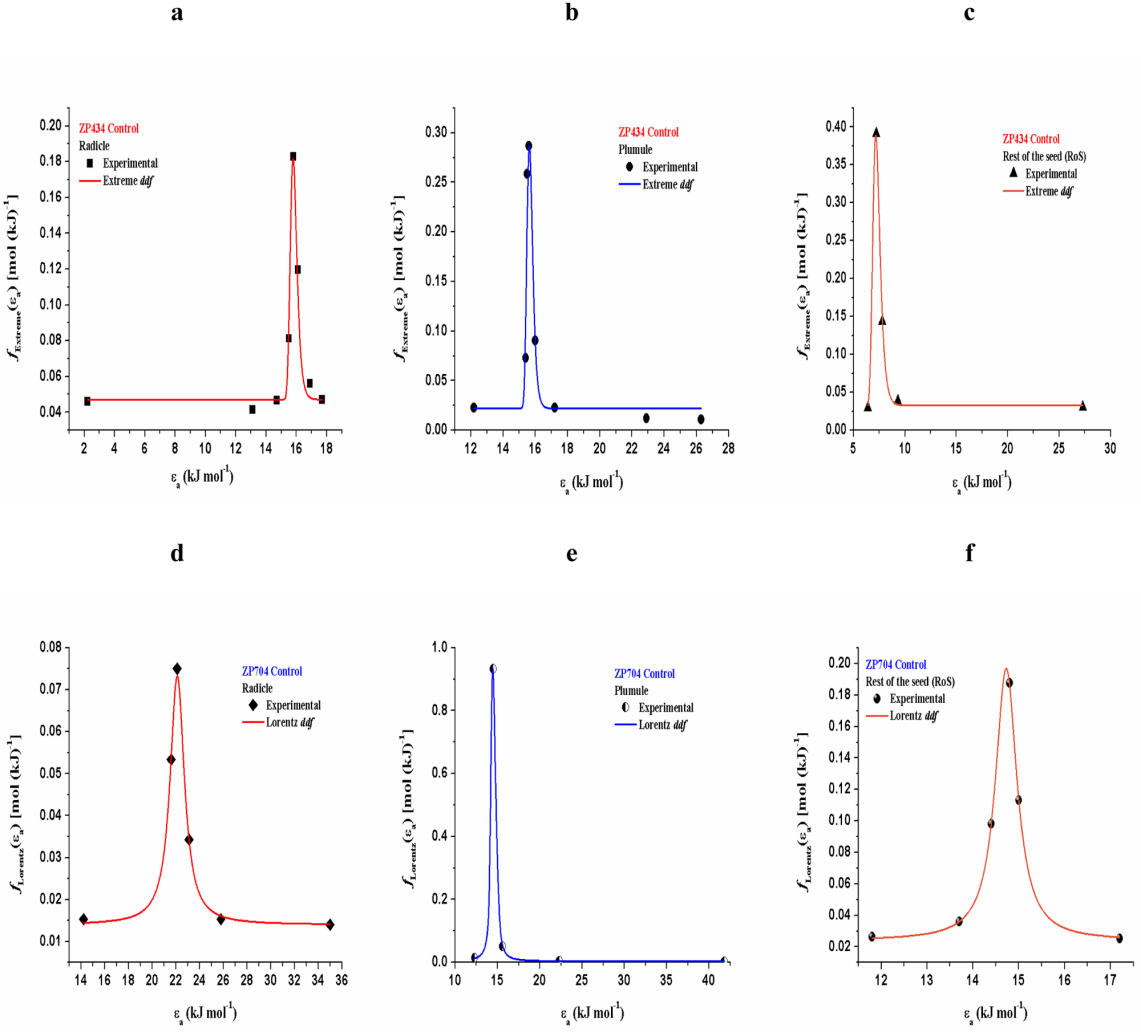
[(a)-(c)—(d)-(f)] Trend of experimental points (not all data points shown) and best correlated density distribution functions of activation energy counterparts for dehydration stress of seedling parts attached to control samples of both tested hybrids.

All seedling parts attached to ZP434 exhibits very narrow extreme (Ex) density distribution functions of activation energy counterparts, with Gumbel (Type I) behavior [34] of random variable (*ε*_*a*_) (Fig 4a–4c). The radicle and plumule dehydration processes are characterized by the smallest extremes stationed at the *ε*_*a*_ value of about 16.0 kJ mol^-1^ (Fig 4a and 4b), while RoS has shifted constrict extreme even to the lower values of *ε*_*a*_, which is located about 7.0 kJ mol^-1^ (Fig 4c).

For ZP704, desorption of water molecules in seedling parts follows a slightly different distributions of activation energy counterparts, which obeys to Lorentz (Cauchy) distributions (Fig 4d–4f), and which are characterized by a slightly wider profiles (except for plumule) than those identified in the case of the ZP434 hybrid (Fig 4a–4c). Spreading the reaction profile in respective distributions may suggest further complicating the dehydration process, where this is especially pronounced in radicle, increasing the *ε*_*a*_ value beyond 20.0 kJ mol^-1^ (Fig 4d). In the case of plumule, we have the opposite behavior, which leads to narrowing of the distribution, and declines in the value of *ε*_*a*_ to about 15.0 kJ mol^-1^ (Fig 4e). For RoS, we have further expansion of distribution which implies an even wider maximum value, which is stationed between 14.5 and 15.0 kJ mol^-1^.

From Table 4 we can see that the onset values of actual distributions for all seedling parts attached to ZP434 control tests are much higher than those present for seedling parts attached to ZP704 control tests, which means that at the probability-time scale we have the presence of certain retention periods in water evaporation within control ZP434 hybrid, where this is not the case with control ZP704 hybrid. This phenomenon is manifested particularly at plumule for control ZP704, with extremely low *f*(*ε*_*a*_)_o_ value, where monitored process takes place quite fast with the increasing rate of dehydration (Table 4). Comparing the same seedling part for observed controls of both hybrids, these events are reflected in the higher value of location parameter for ZP434 (Table 4).

**Table 4:** The distribution parameters related to extreme (Ex) and the Lorentz distributed reactivity profiles for dehydration stress of all observed seedling parts, attached to control tests of ZP434 and ZP704 maize hybrids, respectively. Same table shows the appropriate statistical fitting test analysis. Superscripted “a^”^ represent “The onset of distribution function”, “b^”^ represent “The overall reaction contribution in relation to certain seedling part (normalized into the range [0,1])”, “c” represent”The location parameter”, “d” represent “The scale parameter”, “e” represent “Residual Sum of Squares”, “f” represent “Pearson’s Chi-square (*χ*^2^) test” and “g” represent “Value and error were given with five safe digits in a numeric calculation with 100% succeeds”.

**Control tests—ZP434**			
**Extreme (Ex) density distribution function: *f***_***Extreme***_**(*ε***_***a***_**) = *f*(*ε***_***a***_**)**_**o**_ **+ *ψ* × exp[–exp(–*z*)–*z* + 1]; *z* = (*ε***_***a***_**−*μ*)/*σ***			
**Parameters**	**Radicle**	**Plumule**	**RoS**
***f*(*ε***_***a***_**)**_**o**_ **(mol (kJ)**^**-1**^**)** ^**a**^	0.04736 ± 0.00229 ^**g**^	0.02246 ± 0.00163 ^**g**^	0.03282 ± 0.00631 ^**g**^
***ψ*** ^**b**^	0.34469 ± 0.08389 ^**g**^	0.15475 ± 0.02233 ^**g**^	0.50056 ± 0.13902 ^**g**^
***μ* (kJ mol**^**-1**^**)** ^**c**^	15.74 ± 0.02	15.60 ± 0.02	7.10 ± 0.35
***σ* (kJ mol**^**-1**^**)** ^**d**^	0.15 ± 0.02	0.15 ± 0.02	0.29 ± 0.05
**RSS** ^**e**^	1.02653 × 10^−4^	1.81225 × 10^−3^	4.42845 × 10^−5^
***χ***^**2 f**^	2.56632 × 10^−5^	4.52332 × 10^−4^	4.14905 × 10^−6^
**Control tests—ZP704**			
**Lorentz density distribution function: *f***_***Lorentz***_**(*ε***_***a***_**) = *f*(*ε***_***a***_**)**_**o**_ **+ *ψ* · (2/*π*) × [*σ*/4·(*ε***_***a***_**−*μ*)**^**2**^ **+ *σ***^**2**^**]**			
**Parameters**	**Radicle**	**Plumule**	**RoS**
***f*(*ε***_***a***_**)**_**o**_ **(mol (kJ)**^**-1**^**)** ^**a**^	0.01402 ± 0.00005 ^**g**^	0.00419 ± 0.00023 ^**g**^	0.02391 ± 0.00051 ^**g**^
***ψ*** ^**b**^	0.53760 ± 0.06520 ^**g**^	0.19311 ± 0.03212 ^**g**^	0.26929 ± 0.09569 ^**g**^
***μ* (kJ mol**^**-1**^**)** ^**c**^	22.11 ± 0.01	14.70 ± 0.21	14.73 ± /
***σ* (kJ mol**^**-1**^**)** ^**d**^	1.39 ± 0.04	0.05 ± 0.01	0.56 ± 0.01
**RSS** ^**e**^	1.35879 × 10^−6^	9.75379 × 10^−6^	1.27484 × 10^−6^
***χ***^**2 f**^	6.79395 × 10^−7^	9.01356 × 10^−7^	6.37419 × 10^−7^

**Fig 5:**
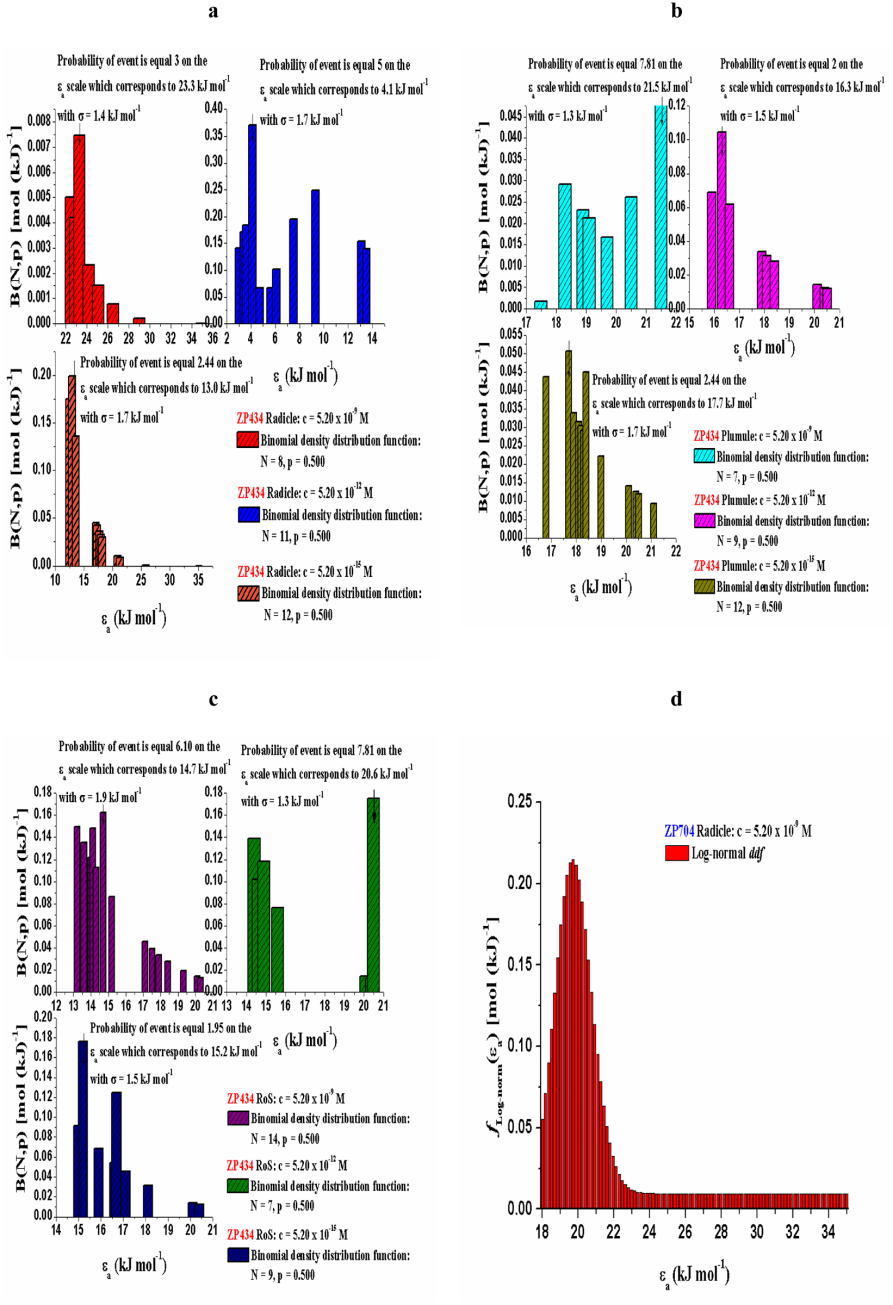
[(a)-(d)] Distribution functions of activation energy counterparts related to dehydration, characterized by typical external features, which are correlated to seedling parts of both hybrids treated with different 24-EBL concentrations (5.20 × 10^−9^ M, 5.20 × 10^−12^ M, and 5.20 × 10^−15^ M).

**Fig 6:**
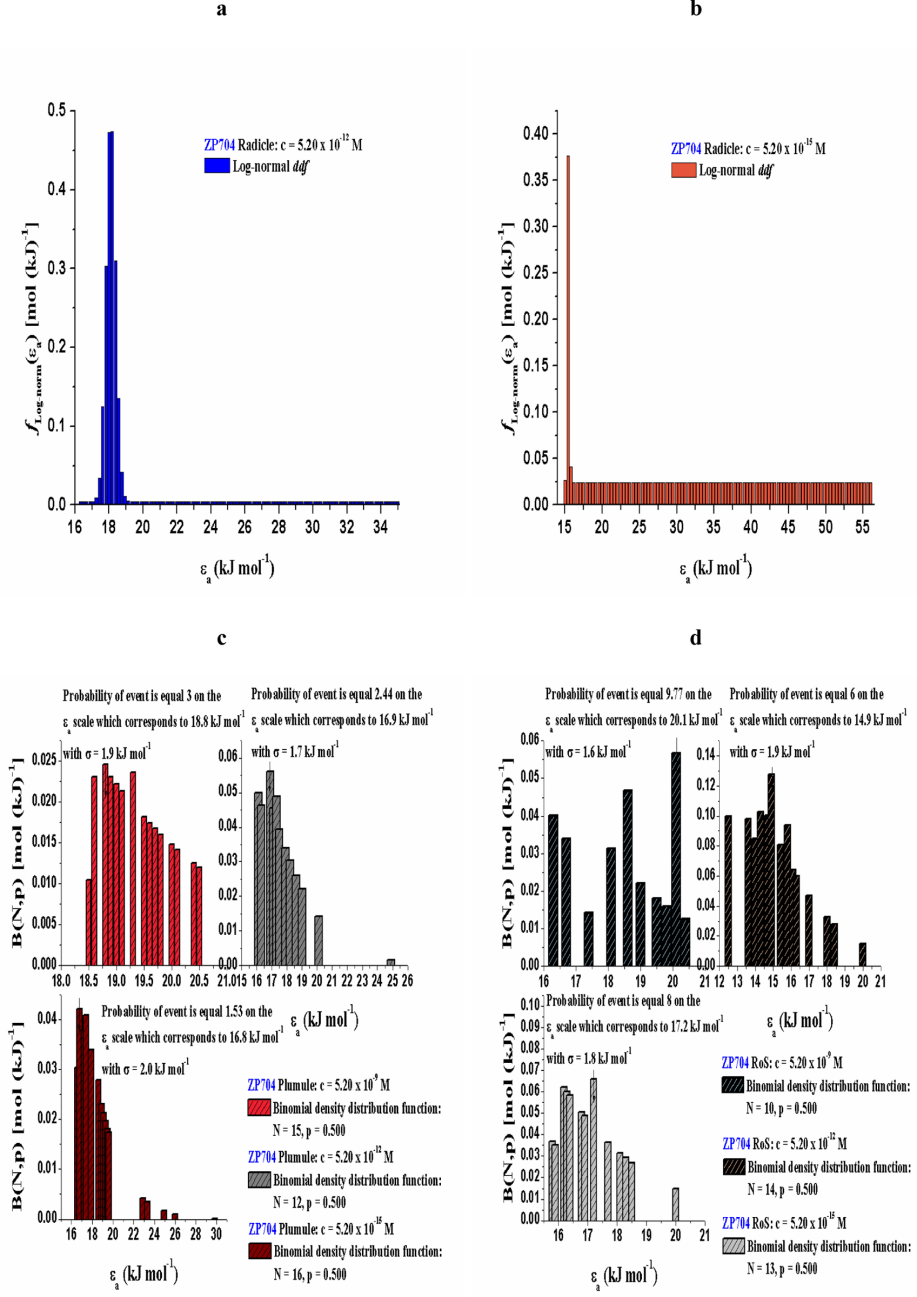
[(a)-(d)] Distribution functions of activation energy counterparts related to dehydration, characterized by typical external features, which are correlated to seedling parts of both hybrids treated with different 24-EBL concentrations (5.20 × 10^−9^ M, 5.20 × 10^−12^ M, and 5.20 × 10^−15^ M).

For ZP434 and ZP704 seedling parts (radicle, plumule and RoS) that were treated with various concentrations of 24-EBL (5.20 × 10^−9^, 5.20 × 10^−12^ and 5.20 × 10^−15^), we can notice quite different behaviors during dehydration (which are manifested by the different distributed reactivity models) (Figs 5 and 6) from those identified in control samples. In actual case, the reactivity distribution exhibits a discrete character in respect to all seedling parts attached to ZP434 maize hybrid.

In the case of dehydration related to radicle attached to ZP704 maize hybrid under the influence of various 24-EBL concentrations, we have a completely different situation. Namely, the desorption reactions occur during the continuous changes of activation energy counterparts, which obeys a continuous distribution of reactivity. The effect of brassinosteroids at the same seedling part to various maize hybrids is reflected in the different nature of water molecules desorption.

In addition, the quantitative variability is the main cause for arising of bell-shaped and symmetrical [Normal] distribution. The current practice in investigation of this type is to use the bars in figures to indicate any deviations from symmetrical behavior and also to indicate the degree of skewness (Figs 5d, 6a and 6b). The model which states the dehydration process in radicle attached to the ZP704 hybrid distinctly genesis of the Log-normal reactivity distribution. This was not the case with the behavior of seedling parts dehydration processes attributed to the ZP434 maize hybrid.

However, the reactivity distributions in the course of dehydration related to plumule and RoS attached to ZP704 hybrid (Fig 6c and 6d) show a discrete character in behavior of activation energy counterparts, as opposed to the one identified in radicle (Figs 5d, 6a and 6b). Hence, the plumule and RoS show quite different behavior during dehydration unlike the radicle. In both considered cases (Fig 6c and 6d), the number of desorption reactions varies (*N* varies within the same probability, with the change of added 24-EBL concentrations (Fig 6c and 6d), where the largest density of energy bars are found (for both cases) within “medium” concentration of 24-EBL (5.20 × 10^−12^ M) with relatively low values of activation energy counterparts.

Table 4 shows that with decreasing of 24-EBL concentration, the mean value of the distribution also decreases. Also, we can observe that with a reduction in concentration of added 24-EBL, the density distribution becomes more asymmetric about mean (Figs 5d, 6a and 6b). On the other hand, Log-normal reactivity distribution shows the lowest mean value (*μ* = 15.5 kJ mol^-1^) (Fig 6b). However, the widest density distribution, and the most activated desorption process in relation to level of *ε*_*a*_ coverage were identified at the highest concentration of 24-EBL (Fig 5d).

In distributed reactivity models established for dehydration stress of considered seedling parts, the pre-exponential factors (*A*_*i*_) depend on the activation energy counterparts (*ε*_*a*,*i*_). These models assume that the discrete distributed reactivity model takes the average pre-exponential factor, which can be observed as a fixed (constant) frequency factor. In numerical computation procedure, in an iterative loop manner, the non-linear regression is used to determine an optimum (average) value of *A*.

**Fig 7:**
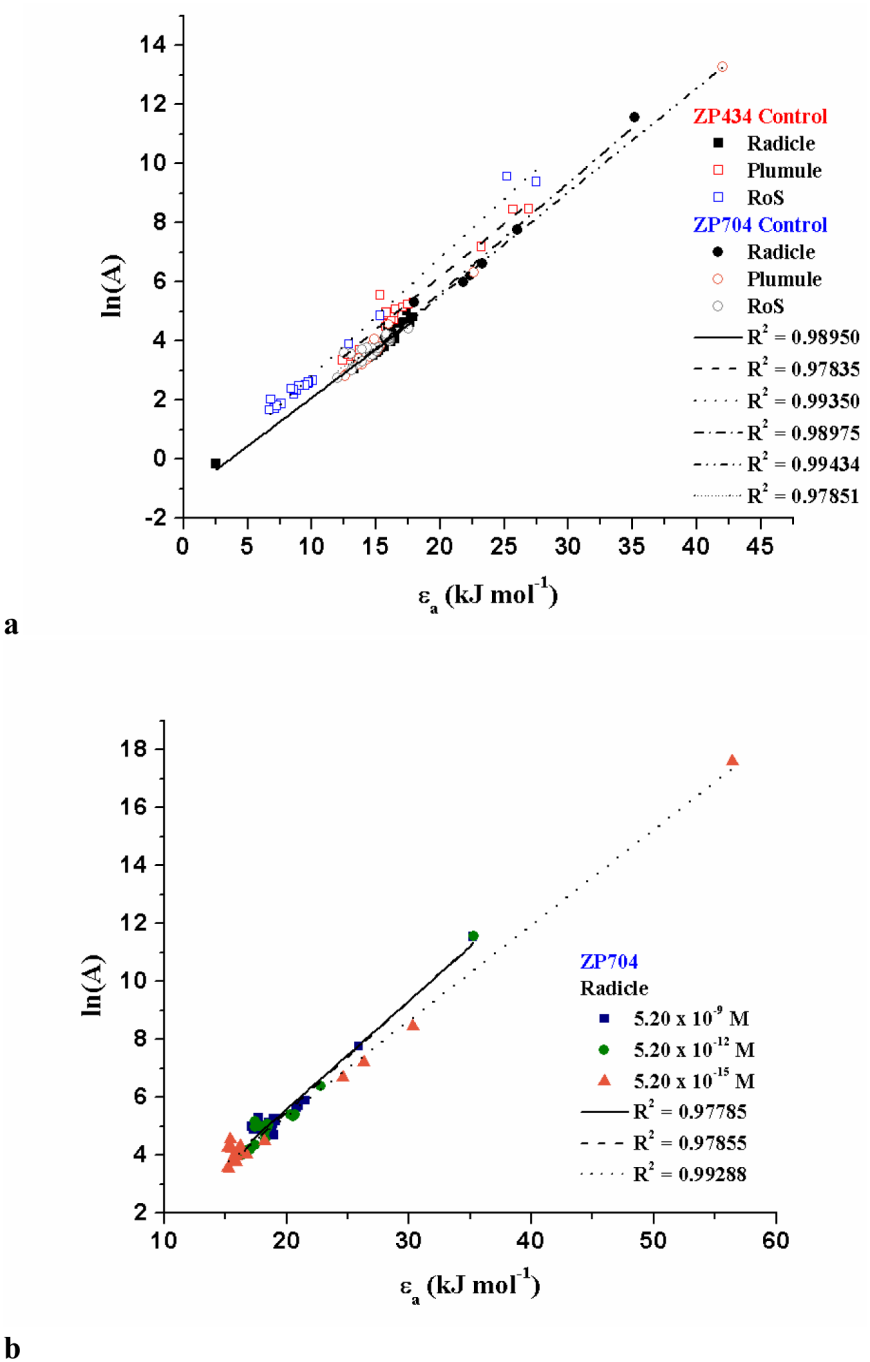
[(a)-(b)] Compensation effect identified for the distributed reactivity models.

Considering Fig 7a and 7b), a positive slopes (*b*) were observed, which correspond to an increasing *A* value (entropy of the activated complex). It should be noted that enthalpy contributions have to be compensated by large entropic contributions to drastically reduce activation energy counterparts (see the range of *ε*_*a*_ values in Fig 7), which requires specific conformational transitions of the macromolecules in the investigated plant systems.

**Fig 8:**
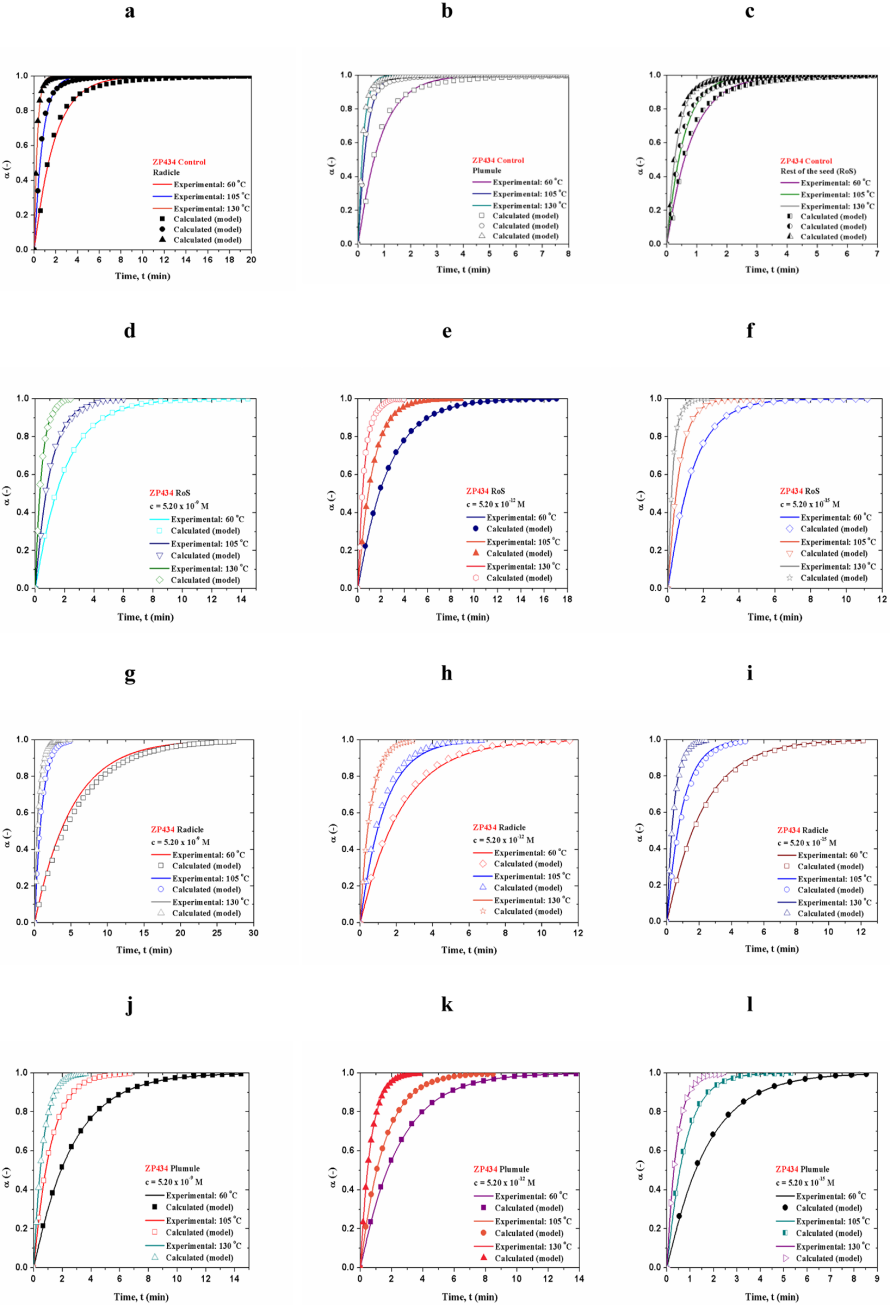
[(a)-(l)] Comparison between experimentally obtained dehydration curves and calculated ones, for ZP434 which were estimated through actual distributed reactivity models using the Eqs (5) and (14).

**Fig 9:**
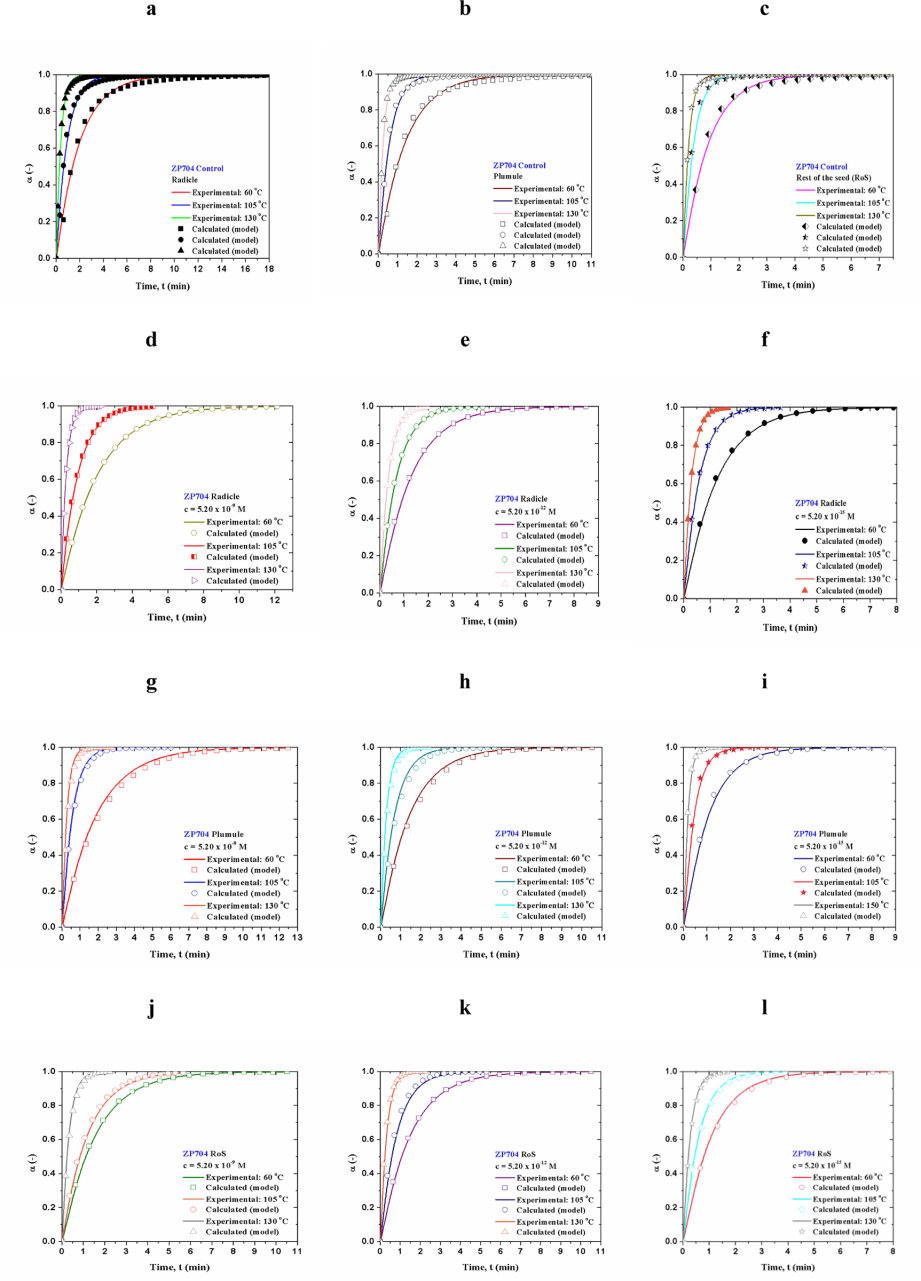
[(a)-(l)] Comparison between experimentally obtained dehydration curves and calculated ones, for ZP704 which were estimated through actual distributed reactivity models using the Eqs (5) and (14).

Figs 8 and 9a–9l show for both hybrids comparison between experimentally obtained dehydration (conversion (α)–*t*) curves and the calculated ones, which were estimated through actual distributed reactivity models, using the Eqs (5) and (14). The magnitude *ψ*_*i*_ in Eq (5) is equal to *ψ* values presented in Tables 4 and 5. The calculated dehydration curves attached to a given distributed reactivity models were designated by the symbols (Figs 8 and 9a–9l). The values of the pre-exponential factors (*A*) which were used in computation procedure at all monitored temperatures are listed in S1 Table (Supporting Information).

**Table 5:** Log-normal distributed reactivity model parameters associated with radicle dehydration stress of ZP704 maize hybrid, under the influence of the different concentrations of 24-EBL (5.20 × 10^−9^, 5.20 × 10^−12^ and 5.20 × 10^−15^ M). Same table shows the appropriate statistical fitting test analysis. Superscripted “a^”^ represent “The overall reaction contributions related to tested radicle dehydration process (normalized into the range [0,1])”, “b^”^ represent “The distribution area”, “c” represent”The mean value”, “d” represent “The standard deviation”, “e” represent “Residual Sum of Squares”, “f” represent “Pearson’s Chi-square (*χ*^2^) test” and “g” represent “Value and error were given with five safe digits in a numeric calculation with 100% succeeds”.

Radicle—ZP704					
***c* = 5.20 × 10**^**−9**^ **M**					
***ψ*** ^**a**^	***A***^**#**^ **(mol (kJ)**^**-1**^**)** ^**b**^	***μ* (kJ mol**^**-1**^**)** ^**c**^	***σ* (kJ mol**^**-1**^**)** ^**d**^	**RSS** ^**e**^	***χ***^**2 f**^
0.51009 ± 0.09125	0.52622 ± 0.05583^**g**^	19.7 ± 0.1	0.052 ± 0.006	7.12918 × 10^−4^	3.56459 × 10^−5^
***c* = 5.20 × 10**^**−12**^ **M**					
***ψ*** ^**a**^	***A***^**#**^ **(mol (kJ)**^**-1**^**)** ^**b**^	***μ* (kJ mol**^**-1**^**)** ^**c**^	***σ* (kJ mol**^**-1**^**)** ^**d**^	**RSS** ^**e**^	***χ***^**2 f**^
0.28021 ± 0.08412	0.35473 ± 0.20372^**g**^	18.1 ± 0.4	0.016 ± 0.005	1.94671 × 10^−4^	6.48911 × 10^−5^
***c* = 5.20 × 10**^**−15**^ **M**					
***ψ*** ^**a**^	***A***^**#**^ **(mol (kJ)**^**-1**^**)** ^**b**^	***μ* (kJ mol**^**-1**^**)** ^**c**^	***σ* (kJ mol**^**-1**^**)** ^**d**^	**RSS** ^**e**^	***χ***^**2 f**^
0.20970 ± 0.06598	0.13667 ± 0.00754^**g**^	15.5 ± 0.1	0.009 ± /	2.58178 × 10^−5^	2.07110 × 10^−6^

From model simulations (Figs 8 and 9a–9l), a very good agreement between all experimentally obtained and all calculated conversion (α–*t*) curves exist (error in curves deviation is less than 1.50%. It can be seen that the derived distributed reactivity model curves describe the actual process without serious deviations.

## Discussion

Comparing the results associated with control samples, we can see that elevated contents of raffinose in RoS within ZP434 in relation to ZP704 hybrid (Table 1) might be likely to higher desiccation tolerance of ZP434 aftermath. Results are in good correlation with assertion that desiccation tolerance in maize can be associated with sucrose-to-raffinose mass ratios less than 20:1 [35].

Significant changes in sorbitol content has not been identified. Namely, sorbitol protects cells from the harmful effects caused by high osmotic pressures and also plays an important role in the process of cell growth, ethanol fermentation, and protein synthesis under heat and ethanol stresses [36]. In addition, trehalose is the most effective carbohydrate in preserving the structure and function of biological systems during dehydration and subsequent storage. Namely, trehalose is the most ejective carbohydrate in conferring protection during dehydration [37]. The efficacy order for membrane preservation is reported to be trehalose, followed by sucrose, and then glucose in a decreasing order [38]. Considering only ZP434 hybrid, the highest content of trehalose was detected in RoS (control sample without added 24-EBL) and in RoS at lowest concentration of 24-EBL, and also in radicle and plumule at the highest added concentration of 24-EBL. Taking into account these results, exogenously added 24-EBL can improve the water status if the considered system under salt stress, and can affect the presence of some essential minerals. Different concentrations of the 24-EBL can improve absorption of K^+^, Ca^2+^, Mg^2+^ and NO_3_¯ [39]. On the other hand, comparing the results for ZP434 with those obtained for ZP704, we can see that the highest content of trehalose is detected for RoS in control and in all seedling parts with the lowest concentration of added 24-EBL (Table 1). The overall values for trehalose in ZP704 were higher than those in ZP434. The extraordinary effect of trehalose may be attributed to creation of hydrogen bonds with membranes or ability to modify the solvation layer of proteins [40,41].

Trehalose has a larger hydrated volume than other related sugars. Trehalose occupies an at least 2.50 times larger volume than glucose and fructose. Consequently, because of this larger hydrated volume, trehalose can substitute more water molecules, and this property is very close to its effectiveness. Therefore, trehalose can have an impact on the level of resistance to thermal activation for dehydration process, and can also be expected to have an impact on a possible change in the reaction mechanism toward stress protection.

On the other hand, the significant changes in the contents of other sugars such as glucose, fructose and sucrose can be observed for seedling parts and control tests for both maize hybrids (Table 1). For plumule (and in addition also for radicle), the contents of glucose and fructose are quite higher for both maize hybrids, which has a direct implication on sucrose content. Very high values are reflected in content of sucrose within plumule comparing both hybrids, especially at an “intermediate” concentration of 24-EBL (5.20 × 10^−12^ M) (Table 1). These results are a direct consequence of increasing ‘building blocks’ of plants, in this respect, referring to growth of the plant. Namely, high carbohydrate availability during heat stress represents an important physiological trait associated with dehydration stress tolerance. Sucrose and its cleavage products regulate plant development and response to stresses through carbon allocation and sugar signaling [42]. In addition, the increase in sucrose contents can also be linked to the increase in starch hydrolysis and synthesis of sucrose. The above facts were confirmed by the results established for principal dimensions (Table 2).

The sucrose may have the function in desiccation tolerance during imbibition and larger oligosaccharides can serve to preserve sucrose from crystallizing [43,44]. Sucrose content can be changed with a fraction of the total soluble-sugar content rather than a pre-conditioning treatment. The higher sucrose content may be needed in imbibing seed for desiccation tolerance. The increase in sucrose (Table 1) in the radicles and plumules is an obvious consequence of the impact on developing of embryos, which coincides with onset of desiccation tolerance of the seeds. Furthermore, sucrose metabolism is involved in responses to environmental stresses in many plant species. In addition, sucrose cannot be used directly for metabolic processes, but must be cleaved into hexoses before entering into the carbohydrate metabolism pathway. Raffinose and sucrose were found in mature axes of the maize and loss of raffinose was found coincident with the loss of desiccation tolerance during their germination [45].

Decreased level of K in radicle and plumule (including control tests) (Table 3) taking into account results for ZP434 hybrid in comparison to ZP704 hybrid, can seriously depress plant resistance to drought stress, as well as potassium absorption. During water stress, radicles regulated their water and ion uptake capacities by modifying PIPs (plasma membrane intrinsic proteins) and K^+^ channel at transcription level to cope with water deficiency [46].

The way in which different spatial location can affect the reactivity of various chemical species inside the plant can be viewed in least two different ways. Firstly, for the conversion that is initiated at the surface (e.g., at a phase boundary), one can expect that the interior chemical specie will convert later on during the process than a surface-bound one.

Alternatively, one can consider the different thermodynamic energies of various spatially-distributed species within observed plant, as those energy variations can likewise affect magnitude of activation energy barrier facing each system species. In either case, the end result is a distribution of activation energy counterparts, which is typically assumed under the condition of constant and the single pre-exponential (*A*) value or the single rate constant value. Approach of distributed reactivity model can properly describe the process in which reflects inherent system heterogeneity that gives rise to a distinctive distribution of activation energy counterparts.

Rapid dehydration increased the sensitivity of seedling tissues to desiccation as indicated by high critical water contents, below which desiccation damage occurred. At the beginning of the process, at an early stage (up to 25% of α’s) (Fig 1a and 1b) (except for RoS in ZP704 (Fig 1b)), in both systems, all seedling parts show a significant sensitivity to thermal stress so that the value of *E*_*a*_ is increased. If we consider only the values for *E*_*a*_ (that do not exceed 20 kJ mol^-1^, in the central part of process) then it could be concluded that their dehydration paths show similar (but not identical) features. Therefore, we could expect similar mechanisms after the establishment of the constancy of *E*_*a*_ values. In the case of ZP704 maize hybrid (Fig 1b), for radicle and plumule dehydration processes, we have a declining trend in values of *E*_*a*_ with α at the beginning of the process, and this trends correspond to the process scheme which includes two different reactivity regions. The first, with faster decreasing of *E*_*a*_ (α ≤ 0.10) values and second, with slower decreasing of *E*_*a*_ (0.10 ≤ α ≤ 0.25) values (Fig 1b). This behavior corresponds to the complex process scheme with a reversible reaction [47]. This scheme may include an endothermic reversible reaction followed by an irreversible one. For such reaction scheme, *E*_*a*_ is limited by the sum of the activation energy of the irreversible reaction and the enthalpy of the reversible reaction at low fraction reacted. With the increases of α, *E*_*a*_ becomes limited only by the activation energy of the irreversible reaction at high α. On the other hand, for ZP434 hybrid system (Fig 1a), the dehydration process of seedling parts shows a somewhat different form of *E*_*a*_—α at the beginning of the same process. This form of *E*_*a*_−α dependency in the early stage of the dehydration process (Fig 1a) may indicate the presence of simultaneous occurrence of the multiple reaction steps complicated with diffusion. For that matter, the *E*_*a*_ values can form the deviate curve dependence which is bent upwards (Fig 1a). Only if one of the two reaction steps is much faster than another, the overall dehydration rate can be determined by the slowest step and the experimental value of effective apparent activation energy becomes the activation energy of actual step (i.e., *E*_*a*_ = *E*_R_ the kinetic-controlled process or *E*_*a*_ = *E*_D_, the diffusion-controlled process, which depends on the ratio of the rate constants of these two kinetic regimes). A reaction with a higher *E*_*a*_ value (Fig 1b) tends to have a weaker interaction with reaction surface and hence will have enhanced mobility that is reflected in larger activation entropy. However, a high frequency of vibration between reaction surface and water molecules implies a strong bond, which results in a higher *E*_*a*_ value, which was observed in the case of dehydration of seedling parts for ZP434 hybrid, in the late stages of the process (Fig 1a). The apparent activation energy can depend strongly on the interactions of water and protein structures. Namely, despite the fact that the polarizability of water is different in the bulk and in the protein, simulations of protein-ligand complexes are mostly carried out in non-polarizable water media. These facts can greatly complicate the release of water from reaction systems which we are studying here, so the process may depend on the presence of intermolecular cation-π, hydrogen bridge (HB) and water bridge (WB) interactions. It should be mentioned, that the amino-acid side-chains that bear a full charge interact particularly strongly with water, even when they form an ion-pairing interaction with another side-chain. Meanwhile, removal of a charged side-chain from water consequently has a large energetic penalty (desolvation energy). The water molecules are polarizable, and they respond to the presence of charged atoms near them. It is important to note, that when the molecular environment surrounding two interacting charges is less polarizable than water, the attenuation of the electrostatic interaction is correspondingly smaller. A charge inside the protein molecule is surrounded by many chemical groups that are not very polarizable (for the instance, aliphatic groups in side-chains) and by some groups that are fairly polarizable (such as the amide and carbonyl groups of back-bone). In contrast to water, which has a very dynamic structure and can readily reorient to interact with charges, atoms in the interior of a protein are relatively rigid and are therefore limited in their ability to attenuate electrostatic interactions. All of the above interactions lead to a larger value of *E*_*a*_, which are observed at the beginning of the process in the case of the seedling parts in ZP704 hybrid, than in the case of identical codes for ZP434 hybrid.

It is obvious that the concentration of 5.20 × 10^−12^ M affects the lowering of energy barrier for the release of water from the system in the case of radicle within ZP434 hybrid as opposed to control sample which was not subjected to a exogenously added 24-EBL (Figs 2, 3 and 4). On the other hand, unlike RoS in control test for ZP434, which is characterized by low values of *E*_*a*_, the hybrid which was subjected to a 24-EBL, RoS shows almost a two-fold higher *E*_*a*_ value. Here, applied 24-EBL increases *E*_*a*_ value, making it difficult the evaporation of water from RoS, unlike the case of the same seedling part, which has not been treated with the phytohormone. Observing the fact that trehalose is the most effective carbohydrate in preserving the structure and function of biological structures during dehydration and subsequent storage, we can conclude that in the case of radicle for ZP434, the *E*_*a*_ value of dehydration is directly proportional to concentrations of the above mentioned sugar (Table 1).

The control sample of ZP434 hybrid has both the lowest value of trehalose and lowest value of *E*_*a*_. Also, in the case of control samples of RoS for ZP434 hybrid, the lowest value of *E*_*a*_ corresponds to the lowest values of trehalose. Trehalose is an effective hydrogen-bond donor and acceptor, and has the highest potential among disaccharides to form hydrogen bonds with biomolecules, and we can assume that in a case of our tested systems, the trehalose has high influence on the dehydration process [48].

In general, *E*_*a*_ represents a measure of plant response to water stress. Apparent activation energy model may investigate plant responses at combinations of a series of water stress conditions and temperatures, and is able to identify relative sensitivity of physiological and biochemical parameters to water stress. An apparent activation energy approach addresses the question of different stress vectors related to desiccation, and can be used to assess cumulative stress (as time function) under desiccation conditions, where the water potential of plant tissue [49] decreases steadily.

### Comprehensive DRM discussion

In the case of RoS for ZP434 (Fig 4c), the strength of desorption centers is weak, so that the water evaporates more easily than in the case of radicle and plumule. In the latter case, there is an increase in asymmetry of distribution, with a pronounced right-oriented tail (Fig 4c). In all cases observed (particularly for RoS), very low values of *ε*_*a*_ at extremes of distributions can be attributed to the activation of protein-surface combinations. Namely, the water molecules probably occupy a greater surface area and therefore have a stronger interaction with biomolecules surface increasing the *ε*_*a*_ value in the case of radicle and plumule dehydrations. On the other hand, for RoS, water does not take enough space on the biomolecular surface, so its ‘interaction’ on the residence-time scale is almost undetectable, i.e. the residence time (*τ*) is extremely low. This phenomenon probably leads to an even greater lowering in the value of *ε*_*a*_. In the cases of radicle and plumule (Fig 4a and 4b), the higher *ε*_*a*_ values at extremes indicates a more water surface dissociation, which gives a more opportunities for favorable interactions (e.g., hydrogen bonding and van der Waals interactions). It should be pointed out that nature of the protein polymer network can affects the water activity, cross-linking reducing the activity, particularly in biological systems. However, the mentioned interactions and the level of water retention can also greatly depend on the heat-soluble protein/sugar fractions and sucrose content. Namely, the amount of water absorbed by protein-sugar mixes may be a function of the sugar content. From Table 1, we can see that the sugar content in seedling parts for control samples of ZP434 hybrid is not the same, where sucrose content dominates in radicle and plumule, explaining that these seedling parts probably absorb much larger amounts of water than RoS, since sucrose may be linked with a “function” of hydrophilic properties of LEA heat-soluble proteins that can control the dehydration at elevated operating temperatures [50]. These facts explain the previously stated allegations.

Compared to cases related to ZP434, we can assume that seedling parts attached to ZP704 (this applies particularly to the radicle and RoS) are much more sensitive to dehydration stress, where actual shapes of distributions may indicate the increased thermal sensitivity of organic compounds in interaction with water. A higher *ε*_*a*_ value leads to higher thermal sensitivity, which is pronounced for radicle attached to ZP704 hybrid (Fig 4d). Namely, the higher *ε*_*a*_ values around the maximum allocation (Fig 4d) indicate that the radicle suffers major changes during dehydration, and more are caused by the operating temperatures than other seedling parts. Namely, the activation energy counterpart may be in a function of seedling parts composition and the water content therein, where diffusion phenomena can have an impact. Obviously, based on the observed distributed reactivity profiles shown in Fig 4, the control tests of ZP704 hybrid exhibit a more complex behavior during dehydration (except the plumule) than those identified in control tests for ZP434 hybrid. Namely, the seedling parts viewed separately by hybrids probably suffered by the different structural alterations within the protein complexes and probably changes the membrane permeability. The dehydration process related to water mobility can be connected with dissociation energy between water molecules and polar molecular groups presented in a given plants. In this regard, it should be stressed that bond dissociation energy is the energy required to break a hydrogen bond, which is in the range of 15.0–23.0 kJ mol^-1^ [51] and this is in a agreement with the results shown in Fig 4.

Comparing results presented in Fig 4c–4f), we may see drastic changes in activation energy counterpart dehydration profiles, where the maximums are situated at about 7.0 and 15.0 kJ mol^-1^, respectively. In RoS, within ZP434 unlike ZP704, the appearance of activation energy counterpart equal to 7.0 kJ mol^-1^ may also be attributed to participation of phenoxyl radicals which arise from enzymatic activity to the interaction of water molecules, creating a phenoxyl radical-water complex [52]. The very low values of *ε*_*a*_ within RoS attached to ZP434 hybrid may also be connected with an OH⋯*π* complex (with the spatial rotation), which is very unstable and briefly living, and such a phenomenon is not observed in the case of ZP704 hybrid.

It should be mentioned that in the absence of brassinosteroid impact, the low *μ* value for RoS in ZP434 control test (Table 4), may be due to the absence of specific short-range interactions (e.g., hydrogen bonding), and this should lead to very small corrugations in the surface energy, which leads to the prevalence of water fast moving.

From the presented results (Figs 5 and 6), we can assume that based on the adsorption/desorption theory for water as a vital part of any living system, in the tested systems, the active surface may exist and must comprise the binding sites available for water adsorption, i.e. the locations to which water molecules can form an adsorption bond. However, this assumption is valid only, if we considered that at time *t* = *t*_*l*_ (where the desorption process is already happening), the overall number of “evaporated” water molecules in the system remains constant during the measurement time. The latter is valid for all situations where the number of “evaporated” water molecules that may “approach” to the surface, is always much larger than the number of available centers at the surface. We may assume that a water molecule occupies a single desorption center only, as well as that they do not interact with each other and desorbed molecules do not dissociate. In actual case, the dehydration process can be described by the monolayer desorption with irreversible first-order kinetics (which is valid for small equilibrium constant), where it assumes the existence of the heterogeneous nature of the desorption centers of the studied systems. Since, that stochastic approach [53] to the single chemical component behaviors (water molecule) can be represented by a binomial distribution, it has been found that the reactivity distribution of the dehydration process of seedling parts covered by ZP434 hybrid can be described by binomial probability density function *B*(*ε*_*a*_;*N*,*p*) in a form:

B(εa;N,p)=CNεapεa(1−p)(N−εa)=CNεa⋅pεa⋅q(N−εa), (9)

where the random variable *ε*_*a*_ is within 0 ≤ *ε*_*a*_ ≤ *N*, and the parameter *N* (*N* > 0) is integer, while the parameter *p* (0 ≤ *p* ≤ 1) represents the real quantity. The parameter *N* represents identical trials (namely, *N* in general designates the size of the statistical sample with *ε*_*a*_ populations), where trials are independent, e.g., each sample “stub” does not affect the others and *P*(“success”) = *p* is the same for each trial. The distribution describes the probability of exactly *ε*_*a*_ successes in *N* trials if the probability of a success in a single trial is *p* (we can also use the tag *q* = (1 –*p*) which represents the probability for a failure, for convenience). In Eq (9), the term:

CNεa=(εaN)=N!εa!(N−εa)!, (10)

represents the Binomial coefficients. The mean number of events is a fundamental property of the stochastic system. This term can be estimated on the classical (without approaching to Bayesian inference [54]) way, so that if the probability *p* is not too large, that the distribution function has significant value for few of high *ε*_*a*_ values, and then the distribution function can be approximated by the Poisson distribution. In this case, the mean number of events (reactions with characteristic value of *ε*_*a*_) can be calculated. In actual case, the set of first-order reactions which represents the desorption process of water molecules from spatially defined centers, may be described by the overall rate-law equation:

$$\frac{d\alpha }{dt}=\sum_{i=1}^{r}k_{i}\cdot {\left(1-\alpha \right)}^{n},$$ (11)

where *n* is the reaction order (where it is assumed *n* = 1). Furthermore, denoting by *P*_*a*_ an adsorbed water molecule having energy *ε*_a_*, the entire process of desorption can be represented by a set of parallel reactions of the first-order given by:

$$P_{a}\overset{k_{i}}{\to }P_{g},\;\left(i=1,2,\ldots ,r\right)$$ (12)

with *P*_*g*_ denoting the same water molecule after desorption or in the gaseous phase. The corresponding rate constants taken in the form:

$$k_{i}=A_{i}\text{ exp}\left(-\frac{\varepsilon_{a,i}}{RT}\right),$$ (13)

are functions of *ε*_*a*,*i*_ and *T*, while *A*_*i*_ represents the pre-exponential factor for *i*-th reaction in a set.

Taking into account Eqs (11) and (13) after separation of variables and re-grouping of obtained terms, the expression for the total conversion fraction (α) in the case of a discrete change of *ε*_*a*_ values, takes a different form in respect to the expression that refers to a continuous change of *ε*_*a*_ values (Eq (5)), so that we finally obtain:

$$\alpha \left(t\right)=1-\text{exp}\left[-\sum_{i=1}^{r}k_{i}\int_{0}^{t}dt\right]\times \sum_{i=1}^{r}\varepsilon_{a,i}\cdot B\left(\varepsilon_{a,i};N,p\right),$$ (14)

where the third term under summation in Eq (14) can be directly replaced by the mean number of events.

In the case of a “discrete” desorption process, which has been observed in 24-EBL treated seedling parts referred to ZP434 hybrid, the number of individually monitored desorption reactions is significantly lower than those identified in control samples of the same hybrid (Fig 4), considering that the treatment with 24-EBL leads to the condition that *N* is not very large, reducing the number of *ε*_*a*_’s, as can be seen in Fig 5a–5c).

For radicle, at the highest concentration of added 24-EBL (5.20 × 10^−9^ M) (Fig 5a) under the discrete reactivity distribution, the largest probability of dehydration event may be observed for a desorption reaction which takes place with the most probable *ε*_*a*_ value of 23.3 kJ mol^-1^ and the coupled dispersion of *σ* = 1.4 kJ mol^-1^. It is obvious that the presence of high concentrations of exogenously added 24-EBL in radicle within ZP434 hybrid leads to the elevated energy of attraction between water molecules and this energy is optimally about 23.3 kJ mol^-1^ [55]. This is the energy required for breaking and completely separating the bond, and equals about half the enthalpy of vaporization (44.0 kJ mol^-1^ at 25°C), as an average of just under two hydrogen bonds per molecule are broken when water evaporates. In addition, the ability of water molecules to form hydrogen bonds with themselves and biological macro-molecules is the single most important parameter to understand structure, function, and regulation of enzymes, genes, and biological membranes. However, often, surface bound water is more structured than liquid bulk water. Thus, the release of many water molecules upon complex formation into the bulk phase increases the entropy of the entire system.

It should be mentioned here that the increased value of activation energy counterparts within radicle dehydration in ZP434 hybrid (Fig 5a) with a high concentration of added 24-EBL at elevated operating temperatures, probably causes the activation of diffusion mechanisms in cutin network and may increase the likelihood of formation of free volumes, large enough to accommodate the diffusing molecule. In this process, the mineral ions may be involved where their intensity of participation depends on the charge. Also, the number of desorption reactions is reduced (*N* = 8, with actual probability of *p* = 0.500, Fig 5a) at high concentration of exogenously added 24-EBL, compared with other lower concentrations. At the added 24-EBL concentrations of 5.20 × 10^−12^ and 5.20 × 10^−15^ M, under equal probabilities, the number of desorption reactions increases (Fig 5a), while the activation energy counterpart’s decreases. Lower concentrations of added 24-EBL allow facilitated dehydration in radicle of ZP434, in this respect that we have freely water vapor diffusion, and these cases are different from the ones observed at high applied 24-EBL concentration. Looking at all of these cases, presumably, the different concentrations of added 24-EBL influence strongly on activation energies for water moving through the radicle within ZP434 hybrid, and brassinosteroid has an impact on the molecular mechanisms of water movement. These differences, cumulatively, could arise from the characteristics linked with self-diffusion or viscosity of water and those associated with the breaking of the hydrogen bond.

In the case of plumule, at all observed concentrations of added 24-EBL (Fig 5b) under the discrete reactivity distribution, the higher values of activation energy counterparts are still retained, and these values can be attributed to the interaction of the water with plumule colloids tissues and this probably results in a higher energy barrier that is greater than for the viscous flow. Lowering the concentration of 24-EBLs causes an increase in *N* value with equal probability. A similar situation can be observed in the case of RoS under the discrete reactivity distribution (Fig 5c), where the influence of variations in the concentration of added 24-EBL had no significant effect on the drastic change of activation energy counterparts. However, specific concentration value attached to added 24-EBL affects the number of desorption reactions, which leads to variations in the value of *N* with the equal probability (Fig 5c).

Taking into account results shown in Figs 5d, 6a and 6b, the number of desorption reactions is growing rapidly, so that they are characterized for each individual observed concentration of applied 24-EBL with a specific value of activation energy counterpart. At all monitored concentrations of exogenously added 24-EBL, the desorption process of water molecules within radicle attached to the ZP704 hybrid can be described by the Log-normal density distribution function (*ddf*) of activation energy counterparts in the form (Figs 5d and 6b):

$$f_{Log-norm}\left(\varepsilon_{a}\right)\equiv f\left(\varepsilon_{a};\mu ,\sigma \right)=\psi \cdot \frac{A^{\#}}{\varepsilon_{a}\cdot \sigma {\left(2\pi \right)}^{\frac{1}{2}}}\times \text{exp}\left[-\frac{1}{2}\cdot {\left(\frac{\text{ln}\varepsilon_{a}-\mu }{\sigma }\right)}^{2}\right],$$ (15)

where the variable *ε*_*a*_ > 0 and the parameters *μ* and *σ* > 0 all are the real numbers. If *u* is distributed as *N*(*μ*,*σ*^2^) [Normal distribution] and *u* = ln*ε*_*a*_, then *ε*_*a*_ is distributed according to the Log-normal distribution. The parameters *μ* and *σ* represent the mean and standard deviation of the variable’s natural logarithm, while *A*^#^ and *ψ* represent the distribution area and the overall reaction contributions related to radicle dehydration stress.

However, there arises the question, which would be the cause of whether we will have the appearance of Normal or Log-normal distribution of activation energy counterparts? One of the most important reasons for occurrence some of these distributions are indication that additive and multiplicative effects give rise to different features. In the considered case, the Log-normal distribution appears when the many small random effects (taking into account the central limit theorem) are generated and where they are multiplicative. These many small random effects can be correlated with micro-disturbing in a space filled with water caused by thermal gradients, increasing the capillary phenomena, and which can induce the thermo-capillary migration.

Structured and undamaged biological materials exhibit a structural hierarchy. The structure and properties manifested at each successive level are dependent on the attributes of elements in the preceding level, the element’s relative concentration, the physical forces involved in their interaction, and manner in which elements are spatially arranged [56].

The current systems mainly consist of the environment which can be described as a watery solution of low molecular weight species, mainly sugars, salts and organic acids and of high molecular weight hydrocolloids, contained in a water-insoluble cellular matrix of macromolecules, mostly carbohydrates, which also include hemicelluloses, proteins and sometimes the lignin. Intracellular air spaces are present in parenchymous tissue and these can be considered as true structural elements, so that they have a strong influence of the perceived biochemical texture. Namely, these constituents are able to interact with water and have the ability to lower its vapor pressure. This is especially pronounced in water moving over “bridge-crossing” between mesophyll cells in the small distances within air space. The small molecules can depress vapor pressure, mainly through the polar binding, whereas the large biopolymers operate through the surface interactions and capillary effects.

Obviously, given the previous discussion related to dehydrations attached to the seedling parts of ZP434 hybrid, there are obvious textural changes of bio-surfaces with which water molecules can interact and thus may lead to changes in the number and even physical characteristics of desorption centers in both studied hybrids. In this way, the investigated maize hybrids obviously react differently to dehydration stress, and this is especially pronounced in the case of radicle, where these differences are most striking. As proof of this fact is the emergence of a different type of distributions of activation energy counterparts as discrete and the continuous reactivity models (Figs 5 and 6). It should be noted that a major contribution to these changes has the presence of various concentrations of 24-epibrassinolide, where attendance of the brassinosteroid in ZP704 hybrid does not adversely affect the behavior of ZP704 hybrid to dehydration stress. This is not the case with ZP434 hybrid, because the presence of various concentration levels of added 24-EBL has a negative impact on the tolerance to dehydration stress. Favorable behavior shows ZP434 hybrid in the absence of 24-epibrassinolide. On the other hand, the presence of an added 24-EBL has a favorable effect on the proper emergence of the plant seeds for tested ZP704 maize hybrid (considering all samples, except control, Fig 1b). It may be noted that used operating temperature range may also have effect on the manners of distributed reactivity models. Namely, the lower operating temperatures, up to approximately 100°C, can be associated to removal of weakly adsorbed hygroscopic water. Thus, higher the rate of water desorption (fraction of water loss at a given *T*) and lower the activation energy counterpart of desorption process being higher the hydrophobicity.

The *ε*_*a*_ values in the range of 14.9–20.1 kJ mol^-1^ (Fig 6c and 6d) can be attributed to diffusion of “desorbed” phosphorous ions through possibly backward water layer (in a short reaction time periods), which can be of great significance in biological processes [57]. The increased activity of phosphorus in such cases could be assumed based on the results shown in Table 3, where a much higher phosphorous content was identified in plumule and RoS under monitored concentrations of 24-EBL attached to ZP704. It should be stressed that emergence of various forms of reactivity distributions and therefore different models of dehydration in seedling parts of maize hybrids is the consequence of different responses of studied systems to the presence of 24-epibrassinolide during abiotic stress, which greatly affects the value of critical water activity related to chemical changes (also associated with the molecular rearrangements which are at the high levels during dehydration stress), including also impact on the glass transition temperature. The glass transition can be related to volatile release. Volatiles can be entrapped within amorphous micro-regions [58] during dehydration of sugar rich systems. This is important in such cases, where volatiles are released when temperature exceeds glass transition temperature [59], due to both temperatures raise and moisture gain, which also lead to structural changes.

In the case of continuous changes of activation energy counterparts, an empirical formula was adapted [60] which assumes a linear dependence between *ε*_*a*_ and logarithm of the activation energy counterpart, as:

$$A\left(\varepsilon_{a}\right)=cons\text{tan}t\times \text{exp}\left(\varphi \cdot \varepsilon_{a}\right)=A_{\mu }\times \text{exp}\left[\varphi \cdot \left(\varepsilon_{a}-\mu \right)\right],$$ (16)

where *φ* is the constant, *A*_*μ*_ is the pre-exponential factor value at the mean value of the estimated density distribution function, *μ* (Tables 4 and 5). With a variable *A*, which follows the changes in *ε*_*a*_ values, the parallel set of first-order reactions was assumed, and this assumption obeys a compensation effect [61] [as ln(*A*) = *a* + *b*·*ε*_*a*_] behaviors.

The existence of the kinetic compensation effects (Fig 7a and 7b) is the obvious consequence of hydrophobic and hydrogen bonding interactions between water and macromolecules, which lead to the enlarging of entropy and enthalpy terms, as was previously assumed.

We can notice two kinetically separated branches, where ln(*A*)–*ε*_*a*_ lines which are almost overlapping at high and “medium” concentrations of 24-EBL (× 10^−9^ and × 10^−12^ M) belong to first branch, while ln(*A*)–*ε*_*a*_ line associated to lower concentration of 24-EBL (× 10^−15^ M) belongs to second branch (Fig 7b). This result suggests that effectively there is a change in mechanism with changing the operating temperature and concentration levels of 24-EBL, for radicle within ZP704 hybrid.

Distributed reactivity models for dehydration stress attached to seedling parts indicate the complex reactivity dependence because of structural changes arising from interactions between water molecules and bioactive compounds present in the plant.

## Supporting information

S1 Table The pre-exponential factors used for computation procedure performed for studied systems. (DOCX)
