# Supplementary material for: New insights in dehydration stress behavior of two maize hybrids using advanced distributed reactivity model (DRM). Responses to the impact of 24-epibrassinolide
Source: PLoS One. 2017 Jun 23;12(6):e0179650. doi: 10.1371/journal.pone.0179650 (PMC5482464; doi:10.1371/journal.pone.0179650)
Supplement: S1 Table — (DOCX) [file pone.0179650.s001.docx]

**Supporting Information**

**PLOS ONE**

**Title: “New insights in dehydration stress behavior of two maize hybrids using advanced distributed reactivity model (DRM). Responses to the impact of 24-epibrassinolide”**

**Authors:**

**Hadi Waisi^1^^[[1]](#footnote-1)^, Bojan Janković^2^, Marija Janković^3^, Bogdan Nikolić^4^, Ivica Dimkić^5^, Blažo Lalević^6^, Vera Raičević^6^**

^1^ Department of Scientific Research and Information Technology, Institute for the Development of Water Resources “Jaroslav Černi”, Belgrade, Serbia,

^2^ Department of General and Physical Chemistry, Faculty of Physical Chemistry, University of Belgrade, Belgrade, Serbia,

^3^Radiation and Environmental Protection Department, Institute of Nuclear Sciences “Vinča”, University of Belgrade, Belgrade, Serbia

^4^ Department for phytopharmacy and Environmental Protection, Institute for Plant Protection and Environment, Belgrade, Serbia,

^5^ Department of Microbiology, Faculty of Biology, University of Belgrade, Belgrade, Serbia

^6^ Department for Environmental Microbiology, Faculty of Agriculture, University of Belgrade, Belgrade, Serbia

S1 Table. The pre-exponential factors used for computation procedure performed for studied systems.

| **Concentration**  **(M)** | **ZP434** | **ZP704** |
| --- | --- | --- |
|  | ***A* (min^-1^)** | ***A* (min^-1^)** |
| **Control**  **Radicle** | 2.470 × 10^2^ | 1.547 × 10^2^ |
| **Control**  **Plumule** | 3.751 × 10^2^ | 1.724 × 10^2^ |
| **Control**  **RoS** | 2.827 × 10^2^ | 3.389 × 10^2^ |
| **5.2 × 10^-9^**  **Radicle** | 5.345 × 10^2^ | 5.526 × 10^2^ |
| **5.2 × 10^-9^**  **Plumule** | 7.120 × 10^2^ | 1.700 × 10^2^ |
| **5.2 × 10^-9^**  **RoS** | 4.156 × 10^1^ | 2.990 × 10^2^ |
| **5.2 × 10^-12^**  **Radicle** | 2.287 × 10^0^ | 5.475 × 10^2^ |
| **5.2 × 10^-12^**  **Plumule** | 1.660 × 10^2^ | 7.550 × 10^2^ |
| **5.2 × 10^-12^**  **RoS** | 4.748 × 10^2^ | 4.770 × 10^2^ |
| **5.2 × 10^-15^**  **Radicle** | 1.880 × 10^1^ | 2.674 × 10^2^ |
| **5.2 × 10^-15^**  **Plumule** | 4.540 × 10^2^ | 5.640 × 10^2^ |
| **5.2 × 10^-15^**  **RoS** | 4.759 × 10^2^ | 6.470 × 10^2^ |

1. *Corresponding author*

   *E-mail address*: [hadiwaisi@yahoo.com](mailto:bojanjan@ffh.bg.ac.rs) (HW). [↑](#footnote-ref-1)
